# Educational attainment in patients with congenital heart disease: a comprehensive systematic review and meta-analysis

**DOI:** 10.1186/s12872-021-02349-z

**Published:** 2021-11-19

**Authors:** Lucia Cocomello, Arnaldo Dimagli, Giovanni Biglino, Rosie Cornish, Massimo Caputo, Deborah A. Lawlor

**Affiliations:** 1 MRC Integrative Epidemiology Unit, Oakfield House, Oakfield Grove, Bristol, BS8 2BN UK; 2 Bristol Heart Institute, Terrell St, Bristol, BS2 8 ED UK; 3 Bristol Medical School, Tyndall Avenue, Bristol, BS8 1UD UK; 4 National Heart and Lung Institute, Imperial College London, London, UK; 5 Population Health Sciences, Bristol Medical School, University of Bristol, Bristol, UK; 6 Bristol NIHR Biomedical Research Centre, Bristol, UK

**Keywords:** Congenital heart disease, Educational attainment, Systematic review

## Abstract

**Background:**

Our aim was to comprehensively review published evidence on the association between having a congenital heart disease (CHD) compared with not, on educational attainment (i.e. not obtaining a university degree, completing secondary education, or completing any vocational training vs. obtaining/completing) in adults.

**Method:**

Studies were eligible if they reported the rate, odds, or proportion of level of educational attainment in adults by whether or not they had a CHD.

**Result:**

Out of 1537 articles screened, we identified 11 (N = 104,585 participants, 10,487 with CHD), 10 (N = 167,470 participants, 11,820 with CHD), and 8 (N = 150,813 participants, 9817 with CHD) studies reporting information on university education, secondary education, and vocational training, respectively in both CHD and non-CHD participants. Compared to their non-CHD peers, CHD patients were more likely not to obtain a university degree (OR = 1.38, 95% CI [1.16, 1.65]), complete secondary education (OR = 1.33, 95% CI [1.09, 1.61]) or vocational training (OR = 1.11, 95% CI [0.98, 1.26]). For all three outcomes there was evidence of between study heterogeneity, with geographical area contributing to this heterogeneity.

**Conclusion:**

This systematic review identified all available published data on educational attainment in CHD patients. Despite broad inclusion criteria we identified relatively few studies that included a comparison group from the same population, and amongst those that did, few adjusted for key confounders. Pooled analyses suggest evidence of lower levels of educational attainment in patients with CHD when compared to non-CHD peers. The extent to which this may be explained by confounding factors, such as parental education, or mediated by treatments is not possible to discern from the current research literature.

**Supplementary Information:**

The online version contains supplementary material available at 10.1186/s12872-021-02349-z.

## Background

Congenital heart defects (CHD) are among the most common types of birth defects, affecting between 6 and 8 per 1000 of live born children [1]. Advances in the management of patients with CHD have enabled substantial improvement in long-term survival even for those with serious cardiac defects [2], with more than 90% of patients with CHD reaching adulthood life [3]. Therefore, the implications of CHD in adult patients have become a key focus of CHD research [4].

An area of particular interest is whether those with CHD have similar educational attainment to their contemporaries without CHD [5]. This is important as higher educational attainment is related to better quality of life, as well as a longer, healthier and disease free life in the general population [6–8], and it is plausible this would also be the case among those with CHD. However, whether educational attainment is lower in CHD patients remains unclear. The different conclusions from individual studies of the relationship between CHD and educational attainment may reflect differences in disease severity between studies as it is plausible that more severe CHD would have a greater impact on educational attainment [9]. As both treatments for CHD, and educational systems and policies, vary across time and between geographic regions it is also plausible that associations might vary by these factors.

The aim of this study was to undertake a comprehensive systematic review and, where appropriate, meta-analysis of all available evidence in order to determine: (a) the association between having a CHD compared with not, on three measures of educational attainment (obtaining a university degree, completing secondary education and completing vocational training) in adults; and (b) if possible with the identified studies, determine whether the associations of CHD with educational attainment vary by disease severity, geographic region and over time. In order to provide comprehensive information for patients, education, and health service providers we included all studies in our review in which the rates, odds, or proportion of any of the three educational outcomes could be obtained in adult CHD patients, irrespective of whether the main aim of the study was to look at the association of having a CHD with educational attainment or not.

## Method

The study was conducted in accordance with the Meta-analyses Of Observational Studies in Epidemiology guidelines for Meta-Analyses and Systematic Reviews of Observational Studies [10].

### Data sources and searches

A comprehensive search of electronic databases MEDLINE and EMBASE was conducted for studies published between the beginning of each database and March 2021 (details provided as Additional file 1). Reference lists of relevant studies were also examined to identify any additional relevant studies not identified in the search.

### Selection of studies and data extraction

All abstracts were screened, and full text assessed for eligibility by two independently reviewers (LC and AD), conflicts were resolved by consensus and, where necessary, through discussion with the other co-authors.

### Eligibility criteria

Original research of any study design that fulfilled the criteria below was eligible; this meant we could include population-based register studies, cohort studies, case control studies, cross-sectional studies, and randomised controlled trials if they included relevant data. We sought studies that included a comparator group of non-CHD patients from the same population for aim (a) (see aims at end of introduction), but we also included studies that only included CHD patients. Whilst these studies may not address the question for patients and their families as to whether they are likely to be as successful in school as their peers, our PPI work suggested it was still helpful to know what proportion of those with CHD obtain a university degree or complete secondary education. Furthermore, we identified a source that provided summary data, stratified by age, of the proportion of people in most countries of the world achieving the three educational outcomes explored in this study (see below). Thus, for most studies that only had data in CHD patients we were still able to compare them to overall educational levels in their country.

Studies were therefore eligible if they reported (or provided sufficient data for us to be able to calculate) the rate, odds, or proportion of level of educational attainment in adults (aged 18 years of age or older) with a history of any CHD. They were also eligible if CHD patients had not undergone procedures, whilst those in which patients had undergone procedures were eligible irrespective of the type, timing, or number of repeat procedures. We also included studies irrespective of whether the aim was to explore educational attainment in patients with CHD or not. The cut-off of 18 years was chosen so that we could assess differences in educational attainment at the age of completion of compulsory education in most high-income countries, and with measures (completing a university degree, secondary education, or vocational training) that are likely to influence future life chances. In initial screening we included studies with a lower age threshold (16 years or older) and, in the data extraction process, explored whether it was possible to obtain results for those only 18 years or older.

### Outcomes

Whether comparing CHD patients to a control group without CHD or comparing the proportion with an educational outcome in CHD patients to country-level proportions, we studied three outcomes, and studies could be included if they had data on at least one of these:

- Obtaining a university degree (including undergraduate and postgraduate degrees)
- Completing secondary education
- Completing vocational training

The outcomes were all analysed as ‘not achieving’ (e.g., not obtaining a university degree).

To avoid double counting data, separate articles reporting educational outcomes in the same patient group were evaluated and the article providing most complete information (largest sample or more recent study) was selected for inclusion.

### Data extraction

Data were extracted independently by two reviewers (LC, AD). For each study, we extracted information on the total number of patients with CHD and those without and, where provided, the number of CHD and non-CHD participants who achieved each educational attainment measure. We also extracted information on the age and sex of participants, the geographical region of the study, year of publication and the severity of the disease. Three authors (LC, RC, DAL) a priori defined key confounders of the association between CHD and educational attainment. Confounders are by definition factors that could plausibly affect the risk of having CHD and the educational outcomes [11]. Maternal pregnancy characteristics (e.g. higher early/pre-pregnancy BMI, smoking and alcohol) have been hypothesised to influence CHD risk in offspring, though whether these are all causal factors for offspring educational attainment is debatable [12]. As these are likely to be influenced by maternal/parental education, which is an important determinant of offspring educational attainment, we considered parental education to be a key confounder. CHD risk also varies by parental age at birth and ethnicity, which in turn influence educational attainment. Therefore, we considered the three key confounders to be parental education, age, ethnicity and extracted information on whether studies adjusted for these. In the risk of bias assessment (see below) we considered a study to have minimal risk of bias from residual confounding if they had adjusted for parental education, ethnicity and age, allowing that adjustment to be for either one, or both, of the parents. All relevant results in whatever form were extracted (i.e., any of: adjusted or unadjusted odds ratios, risk ratios, hazard ratios, differences in risk, with relevant standard errors or confidence intervals, or proportion of participants with each educational measure), with information on what analyses were used to obtain the results.

### Obtaining country level summary data on educational attainment

We extracted summary data from ‘Education at a Glance’ on the proportion of adults (25–64 years old) with each of the three educational attainment outcomes for the country of residence and years of data collection of each included study. Education at a Glance is the authoritative source for information on the state of education around the world [13]. It produces annual reports with the first being published in 1998 and the most recent 2019. The age strata 25–64 years was chosen because it most closely matched the ages across the studies identified in our systematic search.

### Risk of bias assessment

Risk of bias was assessed by two independent reviewers (LC, AD) and disagreements were resolved by discussion with all co-authors.

Risk of bias assessment was performed using the risk of bias instrument for non-randomized studies of exposure [14], which is based on seven items: (1) confounding, (2) selection of participants, (3) classification of exposure, (4) departures from intended exposure, (5) missing data, (6) measurement of outcomes and (7) selection of reported results.

### Statistical analysis

To address patient and family concerns (see Patient and Public Involvement) we quantified (i) educational attainment in patients with CHD compared to their peers without CHD and (ii) educational attainment in CHD patients using all available data.

### Comparing educational attainment in patients with CHD to those without CHD

I. We originally planned to perform the main analysis of the association of CHD with educational attainment by pooling individual study estimates with and without adjustment for prespecified confounders. However, some studies did not control for any covariables and, where they did, most controlled only for age and sex. Only one study controlled for all prespecified key confounders by using a sibling control group. One study adjusted for ethnicity, education and other markers of socioeconomic position and another study parental ethnicity and education. We therefore estimated the pooled odds ratio of not completing different levels of education for CHD patients compared non-CHD controls with and without adjustment only for sex and age. A random effects model (i.e., DerSimonian and Laird) was used to estimate the odds ratios of educational attainment because we a priori assumed that the differences between studies—for example due to differences in terms of which CHDs were included, region of residence of participants and year of study—might influence results. The results from the random effect meta-analyses are the average effects across all different populations. To aid interpretation of the random effects result we calculated prediction intervals, with a method proposed by Higgins et al. [15] based on a t distribution with K-2 degrees of freedom, where K corresponds to the number of studies in the meta-analysis. A prediction interval provides a range within which the potential effect of CHD in any different setting/population will lie, as this may be different from the average effect [16].
  We measured between study heterogeneity using the Cochrane Q statistic and I^2^ and explored possible sources of heterogeneity through subgroup analyses. Our pre-specified subgroup analyses were: proportion of CHD patients with severe disease (≥10% or <10%); year of the study (≥2015 or <2015), geographic region (Europe, North America, Middle East, Asia, Australia), and proportion of females (≥50% or <50%). Exact categories (for geographical regions) and thresholds (for severity and proportion of females were decided after data extraction based on what was feasible and to obtain a similar number of studies (and participants) in each group being compared, where possible. Test for subgroup differences (chi-squared) was used to compare effects between groups.
II. We reported a head to head comparison of between proportions of education attainment reported in CHD patients in studies without a comparison group, and data from the general population using data from ‘Education at a Glance’ (adults aged 24–64 in the country/countries from which the CHD patients came from) [17].

### Estimating the proportion of CHD patients attaining each education level

Finally, we estimated the pooled proportions of CHD patients with each measure of educational attainment across all studies (i.e., both studies that included a non-CHD comparison group and those that did not). Pooled proportions for each outcome of interest (i.e., university, secondary and vocational education attainment) were obtained using the inverse variance method, random effects model (DerSimonian and Laird).

Publication bias was evaluated using funnel plots and Egger's test.

All statistical analyses were performed using R (R Core Team (2019). R Foundation for Statistical Computing, Vienna, Austria. URL https://www.R-project.org/) and meta-R package (Guido Schwarzer (2007), meta: An R package for meta-analysis, R News.

### Patient and public involvement

Prior to analyses, we looked at the work carried out by the CHD charity Little Hearts Matter [18], which works continuously with patients and their family to identify areas of public interest. They indicated education as a key concern for patients and families [19, 20] and this represented a key motivation to undertake this review. At completion of the analysis we met with a group of patients and relatives (two male adult patients, two female adult patients, two mothers of adult patients with CHD) who confirmed that education was a very relevant aspect of their life and a key concern when growing up. In some cases, it was suggested that special educational support could have been useful to them, but this was not provided as not perceived to be necessary by the school. Dissemination of the review’s findings amongst relevant audience (e.g., CHD patients and families, but also teachers) was also recommended.

## Results

**Fig. 1:**
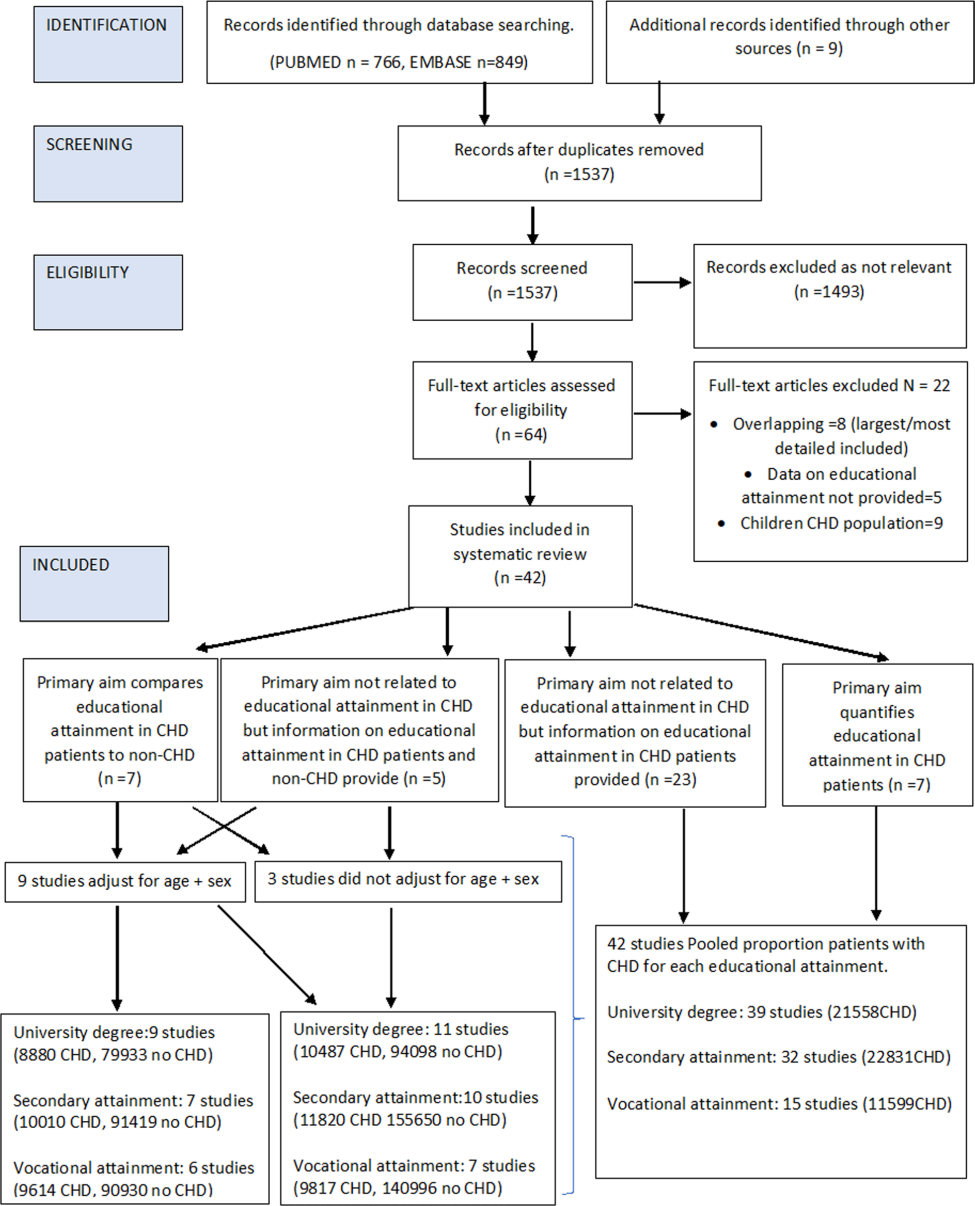
PRISMA Study chart

The titles and abstracts of 1537 articles were screened. Of these, 64 papers were selected and reviewed for inclusion criteria. With detailed review, 22 of these were excluded. Reasons for exclusion were educational attainment not reported (n = 5), overlapping/duplicate studies (n = 8, Additional file 1: Table S1); only children included (n = 9). A total of 42 studies were eligible for inclusion in the review (Fig. 1).

### Characteristics of studies included

An overview of the included studies is presented in Tables 1 and 2. The association of CHD with educational attainment was the main aim for 14 (33% of total) studies [21–34], while in the remaining 28 (67% of total) studies [35–62] it was not. For most of those, information on education attainment was extracted from tables describing study population characteristics. Information on university degree, completing secondary education, and vocational training was available in 39 (93%), 32 (76%) and 15 (36%) studies respectively, with studies able to contribute to more than one outcome. A non-CHD comparison group was included in 12 (29%) of the studies (Table 1) while the remaining 30 reported only on CHD patients (Table 2). The source of the comparison groups varied between studies, but CHD and non-CHD groups were obtained from same underlying population. One study included both a general (unrelated) population comparison group and a sibling (of the CHD patients) comparison group [57]. As none of the other studies had a sibling comparison group we included results from the general population comparison group only in the main meta-analyses and in a sensitivity analysis repeated the meta-analysis with results comparing CHD patients to their siblings.

**Table 1:** Characteristics of studies with a comparison group of people without congenital heart disease

References	Geographic region	Study period	Participants	Educational attainment data source	Sample size	Age of CHD patients (years)	Type of CHD	Factors controlled for	Study aim to assess education outcomes in patients with CHD^a^
Kokkonen [21]	Finland, Europe	–	CHD = individuals born between 1963 and 1968 around Oulu University Central Hospital, who had a diagnosis of CHD Non-CHD = adults selected at random from the population registry of the area	Questionnaire	CHD = 71 Non-CHD = 211	Mean 22.1 (range 19–25)	Mixed CHD	Age	Yes
Simko [35]	US, North America	–	CHD = patients > 18 years of age who were being followed in the outpatient clinic Non-CHD = healthy control peers obtained from a random community sample by “word of mouth,” advertising in churches, supermarket bulletin boards, and the local newsletter	Questionnaire	CHD = 124 Non-CHD = 124	Mean 26.4 (range 18–59)	Mixed CHD	Age, sex, race, and income	No
Rose [36]	Germany, Europe	–	CHD = patients being followed in the outpatient clinic Non-CHD = Samples of the German population collected by different established German opinion research centres	Questionnaire	CHD = 111 Non-CHD = 7355	Mean, SD 33 ± 12	Mixed CHD	Nothing	No
Olsen [22]	Denmark, Europe	2006	CHD = patients with International Classification of Diseases code for CHD in the Danish National Registry of Patients Non-CHD = healthy individuals from Denmark’s Civil Registration System	Denmark’s Integrated Database for Labour Market Research	CHD = 2986 Non-CHD = 29,246	More than 13 years old	Mixed CHD	Age, sex, parental income and education number of siblings, having a single parent	Yes
Ozcan [23]	Turkey, Middle East	2005–2007	CHD = patients who presented to the Impairment Assessment Committee of Military Hospital Non-CHD = healthy peers’ military candidates presented to the same military office	Questionnaire	CHD = 145 Male Non-CHD = 400	Mean 23.8 (range 20–42)	Mixed CHD	Age and sex	Yes
Zomer AC, 2012 (24)	Netherlands, Europe	2009–2010	CHD = patients > 18 years old registered on Congenital Corvita Dutch National Registry Non-CHD = participants from the Utrecht Health Project, dynamic population study	Questionnaire	CHD = 1496 Non-CHD = 6810	Mean 39 (range 29–51)	Mixed CHD	Nothing	Yes
Eslami [37]	Iran, Middle East	2002–2010	CHD = patients admitted to the Tehran Heart Centre and Shahid Rajee Hospital due to CHD Non-CHD = non heart disease participants randomly selected from the same area	Questionnaire	CHD = 347 Non-CHD = 353	Mean 33.2 (range 18–64)	Mixed CHD	Age and sex	No
Caruana [25]	Malta, Europe	2013–2014	CHD = patients being followed in the outpatient clinic Non-CHD = general population from department of health information and research	Questionnaire	CHD = 125 Non-CHD = 372	Mean, SD 30.64 ± 12.80	Mixed CHD	Age and sex	Yes
Rometsch [38]	Switzerland, Europe	2015–2016	CHD = patients being followed in the outpatient clinic Non-CHD = healthy peers identified by the participating patients	Questionnaire	CHD = 188 Non-CHD = 139	Mean 24.7 (range 18–30)	Mixed CHD	Age and sex	No
Udholm [26]	Denmark, Europe	2015–2018	CHD = patients identified using the Danish National Patient Registry Non-CHD = general population from Danish study of Functional Disorders (DanFunD)	Questionnaire	CHD = 140 Non-CHD = 1120	Mean 32.6 (range 18–65)	Unrepaired small ASD	Age and sex	Yes
Schaefer [27]	Switzerland	–	CHD = University Children’s’ Hospital, Zurich Non-CHD = 50,066 General student population of Zurich 2006/2007 served as controls	Questionnaire	CHD = 207 Non-CHD = 38,253	Median 18.58 (range 17–20)	Mixed CHD	Nothing	Yes
Madsen [57]	Denmark	–	CHD = Danish nationwide population-based medical registries Two Non-CHD comparisons = (1) General population cohort identified from Danish Civil register (2) CHD patients’ siblings from same register as (1)	Statistics Denmark	CHD = 7019 Non-CHD = general population 68,805 Siblings 6257	–	Mixed CD	Cohort1: Age and sex Cohort 2: sibling	No

These are the studies included in our main meta-analyses. ^a^We included any study that provided data of the proportion, odds or risk, of patients with one or more of the educational outcomes in CHD patients, irrespective of whether the aim of the study was concerned with educational attainment in CHD patients or not: Yes means the aim at least in part was concerned with educational attainment in CHD patients; No that the aim was not concerned with educational attainment in CHD patients

**Table 2:** Characteristics of studies with no comparison group or with general population comparison but that did not report total number of general populations

References	Study location	Study period	Participant	Educational attainment data source	Age (years)	Type of CHD	Study aims to assess education outcomes in patients with CHD
Otterstad [30]	Norway	1980–1983	CHD = 125 Operated between 1959–1978 at the University Hospital Rikshospitalet	Questionnaire	Mean 42 (range 31–73)	Repair isolated VSD performed after age of 10	Yes
Lillehei [31]	Minnesota	1985	CHD = 105 TOF repair 1954–1960 At the University of Minnesota and Variety Club Hospital	Questionnaire	Range 26–31	Tetralogy of Fallot	Yes
Brandhagen [40]	Minnesota	Examined for CHD 1963 Survey 1989	CHD = 168 Hennepin County Medical Center	Questionnaire	Median 31 (range 24–42 years)	Mixed CHD	No
Moller [33]	Minnesota	VSD operated from 1954 to 1960 Surveyed between 1986 and 1989	CHD = 290 University of Minnesota Hospital	Interview	Range 26–35	VSD	Yes
Ternestedt [42]	Sweden	1985	CHD = 26 Uppsala University Hospital	Interview	Older than 25 years	TOF and ASD should have been operated on before the age of 15 years and be more than 25 years of age at the 20-year follow-up in 1985	No
Nieminen [28]	Finland, Europe	1998	CHD = 2896 patients with surgery between 1953–1989 and registered in the Finnish national research registry of paediatric cardiac surgery Non-CHD = General population statistics, national statistical centre, Statistics Finland. The expected values were calculated as weighted averages of published age- and sex specific rates	Questionnaire	Mean 31.7 (range 18–59)	Mixed CHD	Yes
Kovacs [41]	Canada Florida	–	CHD = 280 Hospital outpatient clinic at the university of Toronto and Florida	Questionnaire	Mean SD 31.9 ± 11.3	Mixed CHD	No
Moons [43]	Belgium	–	CHD = 619 University hospital Leuven	Questionnaire	Mean 24 (range 18–66)	Mixed CHD	No
Chen [48]	Taiwan	–	CHD = 289 National Taiwan University Hospital	Questionnaire	Mean 33.2 ± 10.6	Mixed CHD	No
Riley [45]	United Kingdom	Recruitment 2007–2008	CHD = 99 Outpatient clinic in a specialist hospital in central London, UK	Questionnaire	Mean 37.2 (range 17–67)	Mixed CHD	No
Bygstad [29]	Denmark, Europe	–	CHD = 95 patients operated between 1971 and 1991 at the Aarhus University Hospital Non-CHD = The male age corresponding Danish population from Statistics Denmark,	Questionnaire	Median, IQR 32.2 (18.4–60.0)	Tetralogy of Fallot	Yes
Pike [47]	USA	–	CHD = 54 Ahmanson–University of California, Los Angeles Adult Congenital Heart Disease clinic	Questionnaire	Mean, SD 25.6 ± 9	Fontan	No
Bang [46]	Korea	unknown	CHD = 85 Seoul National University Children’s Hospital	Questionnaire	Mean, SD 26.5 ± 5.9	Mixed CHD	No
Opic [39]	Netherlands, Europe	2010	CHD = 252 Operated between 1968 and 1980 at the Department of cardiology, Erasmus MC Non-CHD = Normative data were specified by sex and age, and were derived from the Dutch Central Bureau of statistics in 2011	Questionnaire	Mean 39.7 (range 35.9–44.9)	Mixed CHD operated before 15 years old	No
Karsenty [34]	France	2013	CHD = 135 Universital Hospital of Toulouse	Questionnaire	Mean,IQR 40 (28–51)	Mixed CHD	Yes
Kahya [44]	Turkey	2008–2012	CHD = 69 Education and Research Hospital, Izmir, Turkey	Questionnaire	Mean, SD 39.7 ± 14.2	Repair ASD	No
O’Donovan [51]	New zeland	2010	CHD = 110 Auckland District Health Board Congenital Heart Disease Outpatients Clinic	Questionnaire	Mean, SD 32 ± 12.85	Mixed CHD	No
Aherrera [50]	Philippines	–	CHD = 92 UP-PGH. Cardiology out-patient clinic	Questionnaire	Mean, SD 32.53 ± 13.58	Mixed CHD	No
Tumin [49]	US	2004–2015	CHD = 426 The United Network for Organ Sharing (UNOS) registry	The United Network for Organ Sharing (UNOS) registry	Mean, SD 35 ± 14	CHD underwent transplant	No
Gleason [52]	USA	–	CHD = 138 CHD who presented for outpatient care at The Children Hospital of Pennsylvania	Questionnaire	≥ 18	Mixed CHD	No
Schiele [53]	USA	–	CHD = 169 Outpatient cardiology clinic at nationwide children hospital and OHIO state university medical centre	Questionnaire	Mean, SD 26.5 ± 7.3	Mixed CHD	No
Pfitzer [32]	Germany, Europe	2015	CHD = 1198 patients born between 1992 and 2011 registered in the Germany National Register for Congenital Heart Defects Non-CHD = General German population, Data in Census 2011 by the Federal Statistical Office Germany	Questionnaire	Mean, SD 30 ± 11	Mixed CHD	Yes
Fedchenko [54]	Sweden	–	CHD = 72 Outpatient clinic Ostra Hospital Gothenburg	Questionnaire	Median 43.5 (range 20–71)	CoA	No
Sluman [55]	International (Belgium, France, Italy, Malta, Norway, Sweden, Switzerland, and The Netherlands (Europe); Canada and the United States (North America); India, Japan, and Taiwan (Asia); Argentina (South America); and Australia)	2013–2015	CHD = 3989 Congenital Heart Disease-international study (APPROACH-IS)	Questionnaire	Median, IQR 32 (25–42)	Mixed CHD	No
Enomoto [56]	Japan	–	CHD = 193 Department of Adult Congenital Heart Disease and Pediatrics, Chiba Cerebral and Cardiovascular Center	Questionnaire	Mean, SD 33.62 ± 10.50	Mixed CHD	No
Connor [62]	USA	2015–2016	CHD = 437 Children’s Hospital, Stanford University	Questionnaire	Mean, SD 32 ± 10	Mixed CHD	No
Martínez‐Quintana [58]	Spain	2017–2018	CHD = 169 Outpatient clinic	Questionnaire	Median, IQR 29 (19–39)	Mixed CHD	No
Steiner [59]	USA	–	CHD = 25 Outpatient clinic	Questionnaire	Median, IQR 38 (21–63)	Mixed CHD	No
Barreda [60]	Chile	2019	CHD = 67 Instituto Nacional del To´rax	Questionnaire	Median, IQR 29 (22–38)	Mixed CHD	No
Soufi [61]	France	–	CHD = 60 Two centre, University Medical Center Jean Minjoz in Besançon and at the Cardiovascular Hospital Louis Pradel in Lyon (France);	Questionnaire	Mean SD 26.7 ± 7.4	Fontan	No

^a^We included any study that provided data of the proportion, odds or risk, of patients with one or more of the educational outcomes in CHD patients, irrespective of whether the aim of the study was concerned with educational attainment in CHD patients or not: Yes, means the aim at least in part was concerned with educational attainment in CHD patients; No that the aim was not concerned with educational attainment in CHD patients

Educational attainment was evaluated with the same method in the two groups, with two [25, 27] exceptions, where information on the control group was obtained by published national statistics.

The number of patients with CHD ranged from 25 to 7019 across the studies. The unadjusted pooled analyses of the association of CHD with educational attainment included 11 (N = 104,585 participants, 10,487 with CHD), 10 (N = 167,470 participants, 11,820 with CHD), and 8 (N = 150,813 participants, 9817 with CHD) for university degree, completing secondary education or vocational training, respectively. Equivalent studies for the age and sex adjusted analyses were 9 (N = 88,813 participants, with 8880 CHD), 7 (N = 101,429 participants, 10,010 with CHD), and 6 studies (N = 100,544 participants, 9614 with CHD) for university degree, completing secondary education or vocational training respectively.

Studies were carried out in Europe (n = 21) [21, 22, 24–30, 32, 34, 36, 38, 39, 42, 43, 45, 54, 57, 58, 61], North America (n = 11) [31, 33, 35, 40, 41, 47, 49, 52, 53, 59, 62], South America (n = 1) [60], the Middle East (n = 3) [23, 37, 44], Asia (n = 4) [46, 48, 50, 56], New Zealand (n = 1) [51] and International (n = 1) [55]. Data on educational attainment were obtained by self‐report questionnaires in the majority (39 (93%)), with the remaining three obtaining this from linkage to national registers [22, 49, 57].

### Comparison of educational attainment between CHD and non-CHD

**Fig. 2:**
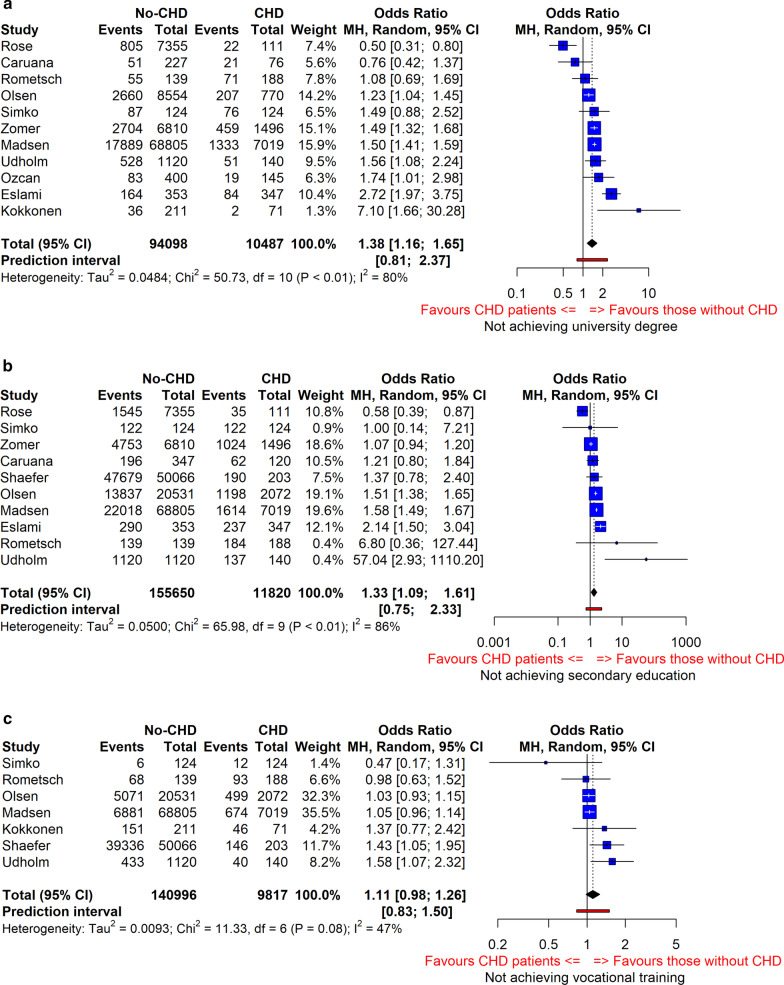
**a** Pooled odds ratio of not achieving university degree comparing CHD patients to those without CHD. **b** Pooled odds ratio of not achieving secondary educational attainment comparing CHD patients to those without CHD. **c** Pooled odds ratio of not achieving vocational training comparing CHD patients to those without CHD

The pooled OR from studies comparing educational outcomes between those with and without CHD showed that patients with CHD had higher odds of not obtaining a university degree (OR = 1.38, 95% CI [1.16, 1.65]) (Fig. 2a), not completing secondary education (OR = 1.33, 95% CI [1.09, 1.61) (Fig. 2b) and not completing vocational training (OR = 1.11, 95% CI [0.98–1.26]) (Fig. 2c). For all three outcomes there was evidence of between study heterogeneity and the predictive interval for the odds ratios were 0.81 to 2.37, 0.75 to 2.33, and 0.83 to 1.50, for not obtaining a university degree, completing secondary education, and completing vocational training, respectively. Similar findings were found in an analysis restricted to the 8 studies that had controlled for sex and age and including the study with siblings as control group (Additional file 1: Fig. S2 and S3a–c).

Subgroup analyses did not suggest that between study heterogeneity was driven by differences in disease severity or year of publication (Table 3). There was some evidence that the increased odds of not obtaining a university degree or completing secondary education was more marked in studies from the Middle East compared to studies from Europe and North America, and that associations for these two outcomes were also stronger in women (Table 3). However, number of studies for subgroup analysis were limited. There was no strong evidence of publication bias (Additional file 1: Fig. S1a–c, Egger's P = 0.74; 0.94; 0.50 respectively for not obtaining a university degree, secondary education and vocational training).

**Table 3:** Subgroup analyses for association between CHD and educational attainment

Subgroup	Number of studies (n CHD cases, n non-CHD)	OR (95% CI) for not achieving educational outcome per subgroup	Test for subgroup differences p value
*Not obtaining a university degree*			
Geographic area			
Europe	8 (9871 vs. 93,221)	1.24 [1.03; 1.49]	0.03
North America	1 (124 vs. 124)	1.49 [0.88; 2.52]	
Middle East	2 (492 vs. 753)	2.29 [1.50; 3.51]	
Year of the study			
Before 2015	7 (3064 vs. 23,807)	1.47 [1.08; 2.02]	0.55
2015 and after	4 (7423 vs. 70,291)	1.30 [1.01; 1.67]	
Proportion of females			
≥ 50%	4 (7630 vs. 70,402)	1.75 [1.30; 2.35	0.14
< 50%	6 (2087 vs. 15,142)	1.17 [0.75; 1.84]	
Proportion of severe disease			
≥ 10%	6 (2376 vs. 8053)	1.49 [1.10; 2.02]	0.43
< 10%	5 (8111 vs. 86,045)	1.25 [0.93; 1.69]	
*Not completing secondary education*			
Geographic area			
Europe	8 (11,349 vs. 155,173)	1.24 [1.01; 1.53]	
North America	1 (124 vs. 124)	1.00 [0.14; 7.21]	0.03
Middle East	1 (347 vs. 353)	2.14 [1.50; 3.04]	
Year of the study			
Before 2015	5 (4150 vs. 35,173)	1.21 [0.87; 1.67]	0.34
2015 and after	5 (7670 vs. 120,477)	1.52 [1.08; 2.14]	
Proportion of females			
≥ 50%	4 (7630 vs. 70,402)	1.88 [1.22; 2.89]	0.04
< 50%	5 (2118 vs. 64,717)	1.01 [0.74; 1.40]	
Proportion of severe disease			
≥ 10%	6 (2478 vs. 57,839)	1.39 [0.99; 1.94]	0.77
< 10%	4 (9342 vs. 97,811)	1.30 [1.02; 1.67]	
*Not completing vocational training*			
Geographic area			
Europe	6 (9693 vs. 140,872)	1.12 [1.00; 1.25]	
North America	1 (124 vs. 124)	0.47 [0.17; 1.31]	0.10
Middle East	–	–	
Year of the study			
Before 2015	3 (2267 vs. 20,866)	1.03 [0.74; 1.43]	0.43
2015 and after	4 (7550 vs. 120,130)	1.20 [0.96; 1.51]	
Proportion of females			
≥ 50%	3 (7283 vs. 70,049)	1.10 [0.73; 1.65]	0.52
< 50%	3 (462 vs. 50,416)	1.28 [1.02; 1.61]	
Proportion of severe disease			
≥ 10%	3 (515 vs. 50,329)	1.04 [0.65; 1.67]	0.87
< 10%	4 (9302 vs. 90,667)	1.08 [0.97; 1.20]	

The proportions with each educational outcome by country, in studies that did not report a peer non-CHD group, are compared to the summary data from ‘Education at a Glance’ in Table 4. For the majority, the proportions of each outcome in CHD patients were similar to the country level data for adults.

**Table 4:** Educational attainment for adult (> 18 years) CHD patients compared to educational attainment in all adults (25–64-year) from the same country as the CHD patients using data from ‘Education at a Glance’

References	Country	CHD patients			Whole country		
		University degree %, [95% CI]	Secondary education %, [95% CI]	Vocational training %, [95% CI]	University degree (%)	Secondary education (%)	Vocational education (%)
Ternestedt [42]	Sweden	27 [12–48]	81 [61–93]		32	81	
Nieminen [28]	Finland	10 [9–11]	78 [77–80]		34	76	
Moons [43]	Belgium	42 [38–46]	98 [96–99]	35 [31–39]	33	70	2
Kovacs [41]	Canada/US	61 [55–67]			41	89	
Riley [16]	United Kingdom	58 [47–67]			38	75	
Ozcan [23]	Turkey	13 [8–20]			14	32	
Bygstad [29]	Denmark	31 [21–41]	68 [58–78]	27 [19–37]	34	77	
Pike [47]	US	61 [47–74]			42	89	
Bang [467]	South Korea	85 [75–92]	95 [88–99]		41	82	
Opic [39]	Netherland	27 [22–33]	74 [68–79]		36	77	0
Karsenty [34]	France		38 [30–47]		34	78	0
Eren [44]	Turkey	19 [10–30]	54 [41–66]		46	91	
O’Donovan [51]	New Zealand	26 [18–36]			42	78	
Tumin [49]	US	51 [46–56]			47	91	
Schiele [53]	US	36 [29–44]			48	91	
Fedchenko [54]	Sweden	50 [38–62]	93 [85–98]	12 [6–22]	43.3	83.2	7.4
Pfitzer [32]	Germany		46 [42–49]		29.1	86.7	12.2
Enomoto [56]	Japan	58 [51–65]			52	100	
Connor [62]	US	50 [46–44]	100 [99–100]		47.4	90.8	0.4
Gleason [52]	US	59 [50–68]	100 [97–100]	6 [4–8]	47.4	90.8	

Education at a Glance population sample size not reported; data are presented in broad age groups and the 25–64 year old group was the one that matched best with the main age of participants across our studies

### Proportions of CHD patients with each educational outcome

The pooled proportion of patients with CHD who completed a university degree, secondary education and vocational training was 36% [95% CI 30–43], 84% [95% CI 76–90] and 25% [95% CI 16–36] across all studies (Additional file 1: Fig. S4a–c). There was substantial between study heterogeneity and the predictive interval was 0.08 to 0.78 for obtaining a university degree, 0.23 to 0.99 for completing secondary education and 0.03 to 0.75 for completing vocational education.

### Risk of bias

The item most identified at risk of bias was confounding, due to parental ethnicity, education, or age, as studies either controlled only for patient age and sex or nothing (Additional file 1: Table S2).

## Discussion

The main finding of the present systematic review is that, despite patients and parents identifying educational attainment as a key concern, there is a paucity of research on the relationship of having a CHD and educational attainment. With an extensive search we identified only 12 studies with a comparison group of people without CHD, with only one adjusting for key confounders such as parental education, ethnicity, and age. Our meta-analysis of these studies showed a trend toward lower odds of completing a university degree, secondary education, or vocational training. However, given the sparsity of studies and between study heterogeneity the predictive intervals for all outcomes suggested educational attainment could be importantly lower or higher in those with CHD compared to their peers.

Despite havingcompiled all available published data since 1986 on university degree, secondary education, and vocational training in CHD patients, we found a very limited number of studies addressing this subject. Pooling evidence from studies that included a control group we found that patients with CHD were at higher odds of not completing university, secondary and vocational educational levels compared to non-CHD peers. There was evidence that this gap was more pronounced in studies from the Middle East compared to those from Europe and North America. It is likely that different educational systems might have a different impact on educational attainment among children with CHD. These aspects may include curricula, methods of teaching, access to teaching material, and the quality and extent of special educational support offered to children who might have reduced school attendance due to repeat treatments. Studies included in the present meta-analysis did not report information on educational support. However, a previous report has shown that in North America children with CHD are more likely to receive additional educational support compared with their peers [63]. We also found some evidence that the gap in education attainment may be more pronounced in females. This could possibly reflect the fact that in general girls do better in school than boys, and additional needs may therefore be less apparent in girls with CHD. However, it is important to note that we have limited statistical power for any of our subgroup analyses.

It has been hypothesised that children with CHD may be exposed to neurotoxic factors which can affect brain development, such as cyanosis, neurotoxicity related to the use of cardiopulmonary bypass, and hypothermic circulatory arrest in children undergoing heart surgery ration [64]. These concerns have prompted recent improvements in surgical techniques and patient management, including the adoption of neuroprotective strategies [65]. The extent to which these would redress the lower educational attainment in patients with CHD is as yet unknown. It is also possible that chromosomal abnormalities commonly associated with CHD, such as Down’s syndrome, contribute to lower educational attainment in CHD patients [66]. It was not possible to determine the extent to which these abnormalities explained the results of our systematic review and it would be useful for future studies to attempt to explore this, for example, by stratifying results by chromosomal abnormality and/or undertaking sensitivity analyses with this group removed. Chromosomal abnormalities may be related to more severe CHD and the odds ratios for not attaining different educational outcomes were higher in the subgroup of studies including more severe CHD patients, but we had limited power to detect differences between the two subgroups based on severity.

The incidence of psychological and psychiatric disorders such as inattention and hyperactivity have been reported to be high in CHD patients [67] and these conditions affect academic performance [68]. Again this was not explored in studies that we identified for this review. Finally, patients with CHD are likely to experience recurrent chest infection [69, 70], endocarditis, cardiac arrhythmias [71] or repeated surgeries with frequent and prolonged absence from the school. However, the studies we identified and reviewed did not explore whether associations were mediated by school absence. Whilst some studies selected people without CHD from general population registers, others recruited from peers or friends of the CHD patients or their families, through media adverts or people with other disorders. One recent large record-linkage study that aimed to compare attainment of self-sufficiency and other outcomes, including educational attainment, among CHD patients and those without CHD undertook within sibling and general population analyses [57]. In our main analyses we pooled results from the general population comparison group (consistent with other studies included in the meta-analysis) but we also repeated the analysis with the odds of each education outcome in CHD patients versus their siblings, and we found very similar results to the general population comparison. Within sibling comparisons such as this are able to control for unobserved fixed family confounding, such as parental ethnicity, socioeconomic position and education [72]. Thus, these findings provide some support that the overall meta-analysis results may not be majorly affected by key family confounding but as this is one single study the potential for residual confounding to have influenced our findings should still be considered.

There were differences between findings from meta-analyses of the 12 studies that had included a comparison group and those studies combined with an additional 30 that only included adults with CHD. In the studies with a non-CHD comparison group we found evidence of lower educational attainment in CHD patients, though with substantial between study heterogeneity. By contrast, when we compared the proportion of educational attainment in CHD patients reported by studies included with data in the national population from Educational at glance when available, we did not find any strong differences. The latter results are limited by lack of adjustment for any potential confounders, and the inclusion of the whole national population in the comparison group, including those with CHDs. Furthermore, whilst we tried to match the year of data collection in the CHD study as close as possible to the year of national data collection, differences in education policy and provision over time may have impacted results, which may have contributed to similar results between the two groups.

A key result of our review is to highlight the paucity of high quality research in this area. On the basis of evidence from studies that have included a non-CHD comparison group we would suggest that training programmes for school personnel and additional educational support for students with CHD should be considered.

### Strengths and limitations

The key strength of this study is our attempt, for the first time, to obtain and review all relevant data, including studies where the aim was to assess the association of having a CHD with educational attainment and those where this was not the aim. We acknowledge for the latter that our search strategy may have missed some studies where a description of educational attainment in patients with CHD was somewhere in the paper. We have presented predictive intervals, as well as odds ratios and confidence intervals, which are recommended when undertaking random effects meta-analyses because of assumed between study heterogeneity, but rarely undertaken [16, 73]. We attempted to standardize academic levels achieved whilst focusing on key measures that are related to future employment, socioeconomic position and health (university, secondary and vocational training). However, we acknowledge that across different educational systems the level of knowledge and skills required is likely to vary. Our results are limited by the sparsity of studies and the lack of any studies that have controlled for key confounding factors.

### Challenges of undertaking research in this area and some possible opportunities

Research in this area is affected by the rarity of the conditions, which limits the possibility to undertake a robust analyses within the single birth cohorts. On the other hand, linkage between educational and health data has not been systematically performed across all countries. Despite recent advances in multidimensional data repositories, which may facilitate research in this area, large registries are unlikely to allow discrimination between the large spectrum of different types of CHD and their different impact on neurological development and educational attainment. Large birth cohort collaborations such as LifeCycle [74] can potentially offer the advantage of achieving a larger sample of patients with CHD [12] with granular longitudinal data and the possibility to investigate variability related to different countries and educational systems.

## Conclusions

In conclusion, in the present systematic review and meta-analysis we appraised current literature on educational attainment in patients with CHD. We found that there is a limited number of studies addressing this topic and the majority of them are limited by lack of comparison group and adjustment for key confounding factors. Bearing in mind these limitations, our analysis showed some evidence of lower educational attainment in CHD patients. Further studies are of paramount importance, with large collaborations across birth cohorts being one potential mechanism for improving research in this area.

## Supplementary Information

**Additional file 1:** Supplementary material.

## Data Availability

All data generated or analysed during this study are included in this published article and its supplementary information files.

## Abbreviation

CHD: Congenital heart disease
