# Supplementary material for: Educational attainment in patients with congenital heart disease: a comprehensive systematic review and meta-analysis
Source: BMC Cardiovasc Disord. 2021 Nov 19;21:549. doi: 10.1186/s12872-021-02349-z (PMC8603574; doi:10.1186/s12872-021-02349-z)
Supplement: Supplementary file 1 — Additional file 1: Supplementary material. [file 12872_2021_2349_MOESM1_ESM.docx]

**Supplementary material 1 method:**

**Data sources and searches**

A comprehensive search of electronic databases MEDLINE and EMBASE was conducted for studies published between the beginning of each database and March 2021. On the 6^th of^ April 2020 and on the 24^th of^ March 2021, we conducted a search using the following strategy:

1) Medline: ((congenital heart disease [TIAB]) OR (congenital heart defect [TIAB]) OR (congenital heart malformation [TIAB])) AND (education (TW) OR (academic performance [TIAB]) OR (academic achievement [TIAB]) OR (school level[TIAB]) OR (educational attainment[TIAB]) OR (quality of life[TIAB]) OR (employment[TIAB]));

2) EMBASE: Search 1: congenital heart disease, ab,ti Search 2: congenital heart defect, ab,ti; Search 3: congenital heart malformation, ab,ti; Search 4: 1 OR 2 OR 3; Search 5: quality life, ab,ti; Search 6: academic performance, ab,ti Search7: academic achievement, ab,ti Search 8: school level, ab,ti Search 9: educational attainment, ab,ti Search 10: education, ab, ti, tw Search11: employment, ab, ti Search 12: 5 OR 6 OR 7 OR 8 OR 9 OR 10 OR 11 Search 12: 4 AND 12

**Supplementary Figure 1a Funnel plot of studies included in pooled analyses of odds ratios of university degree.**


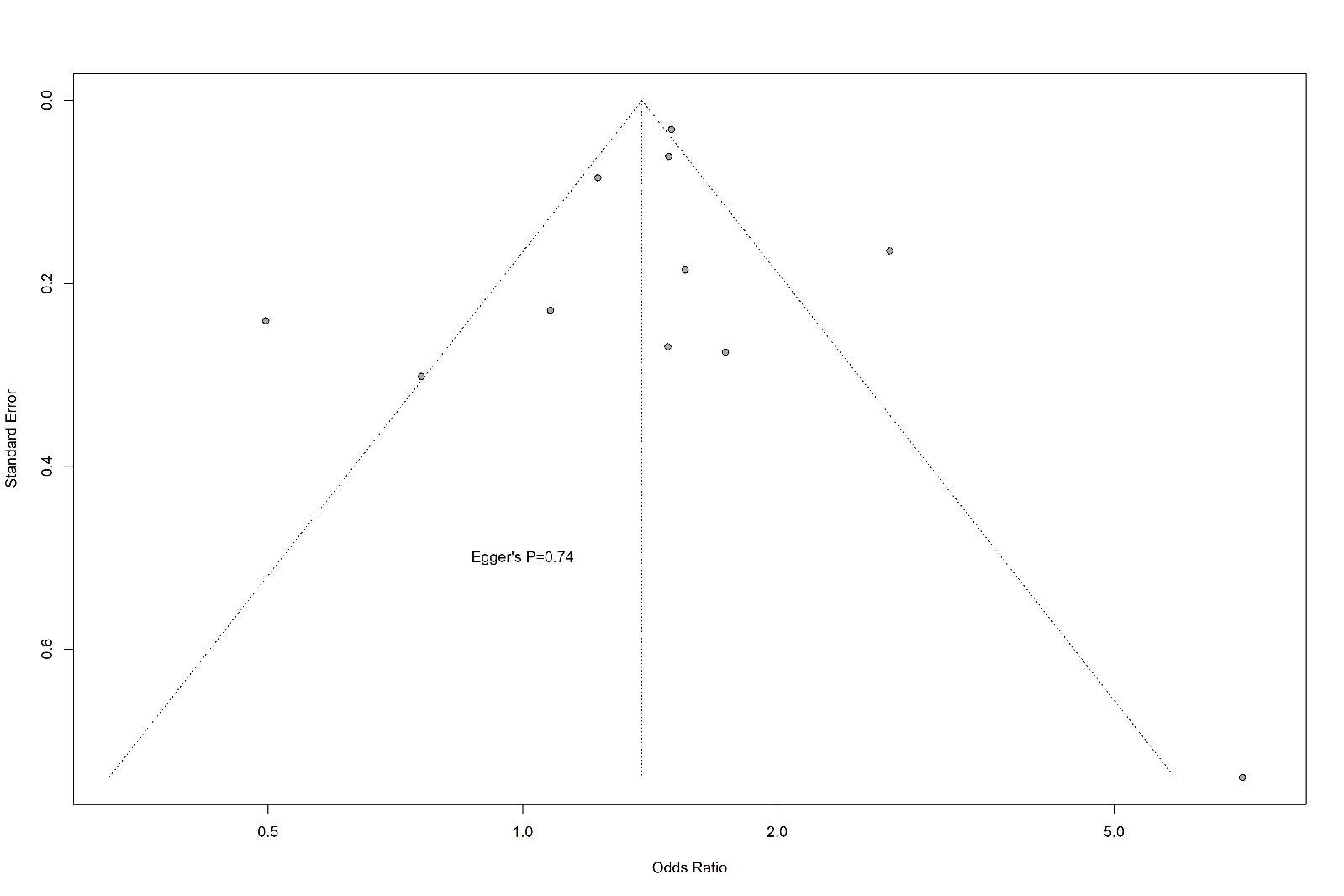


**Supplementary Figure 1b Funnel plot of studies included in pooled analyses of odds ratios of secondary educational attainment.**


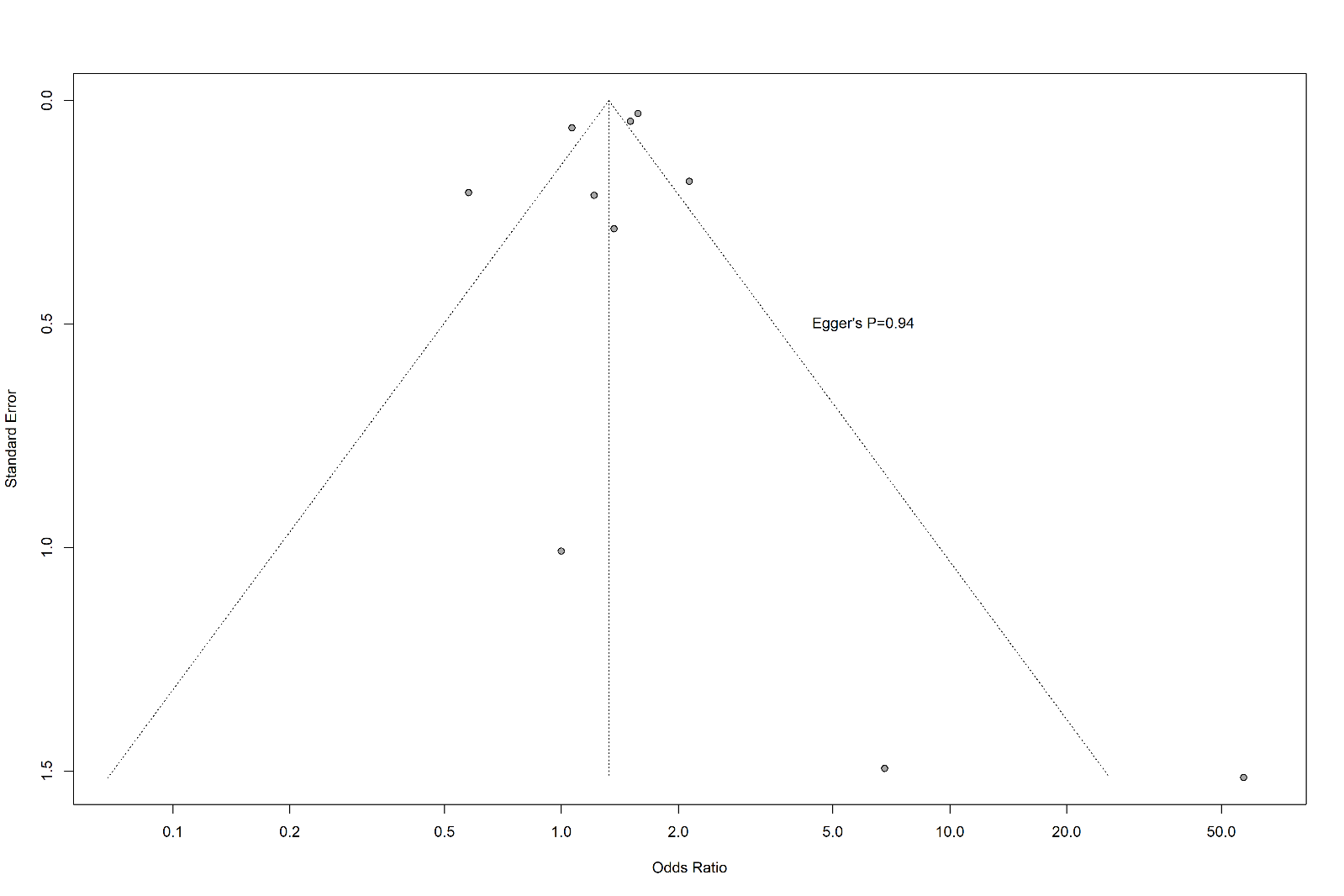


**Supplementary Figure 1c Funnel plot of studies included in pooled analyses of odds ratios of vocational training.**


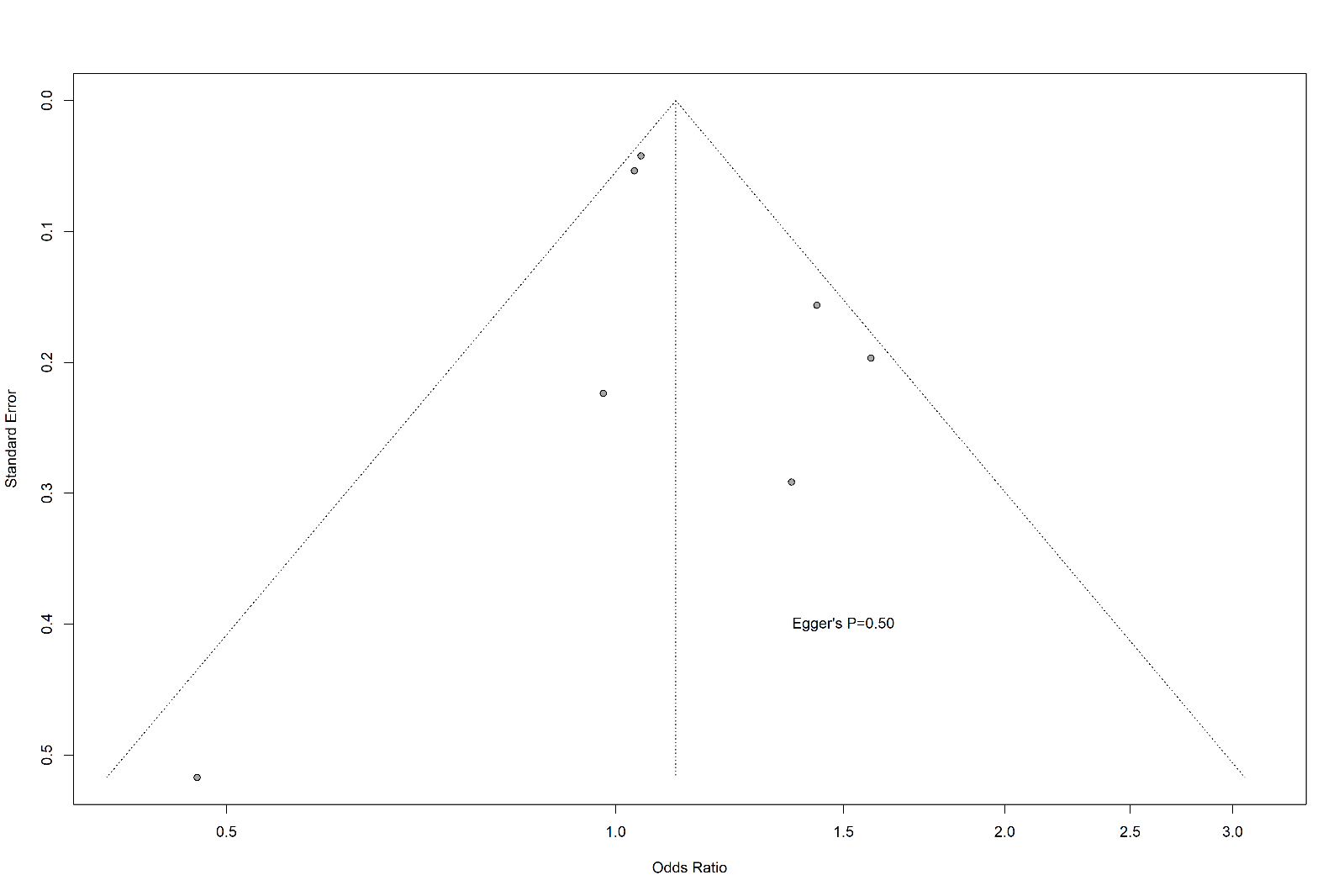


**Supplemenatry Figure 2a** **Pooled odds ratio of not achieving university degree comparing CHD patients to those without CHD with control for age and sex**


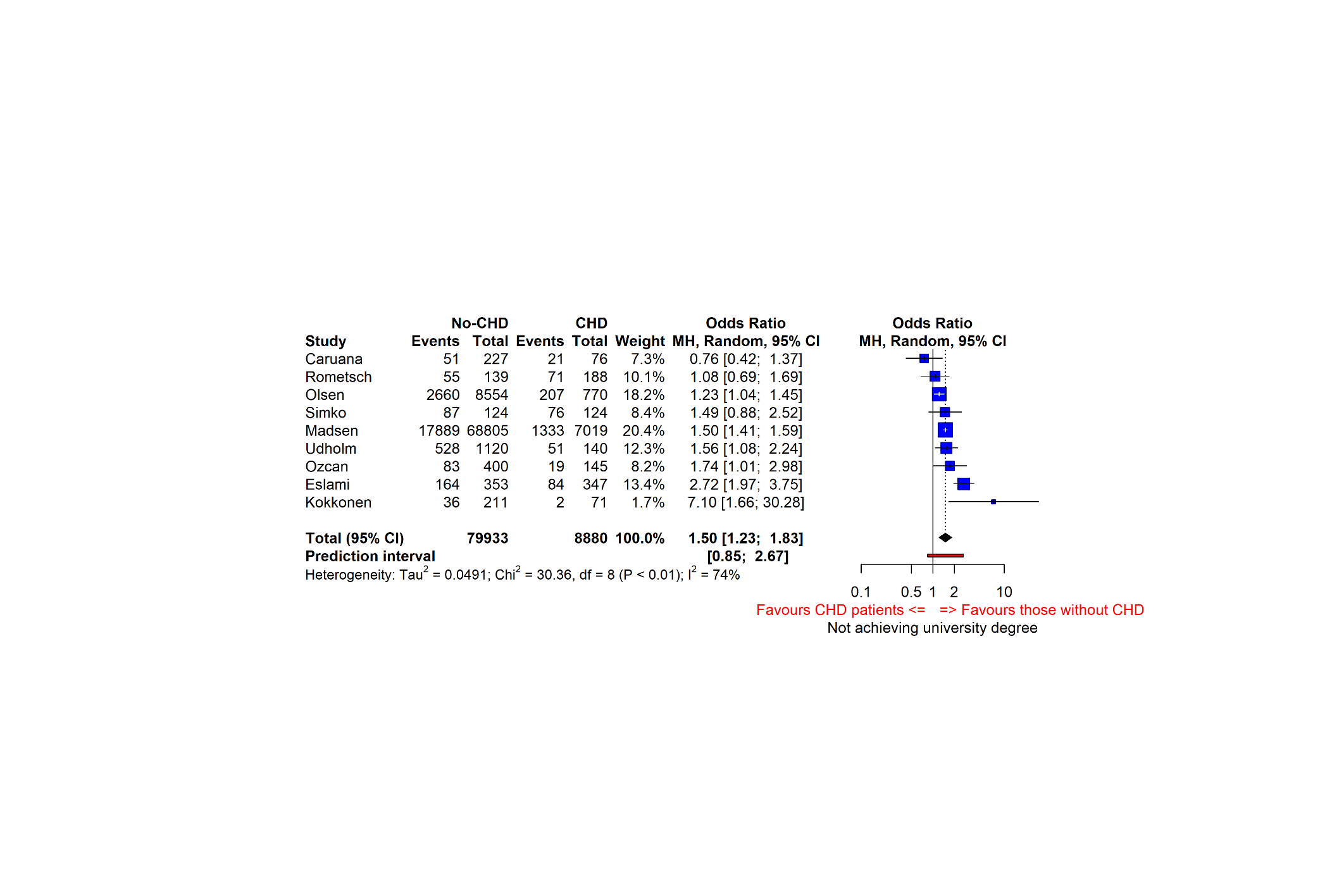


**Supplementary Figure 2b** **Pooled odds ratio of not achieving secondary educational attainment comparing CHD patients to those without CHD with control for age and sex**
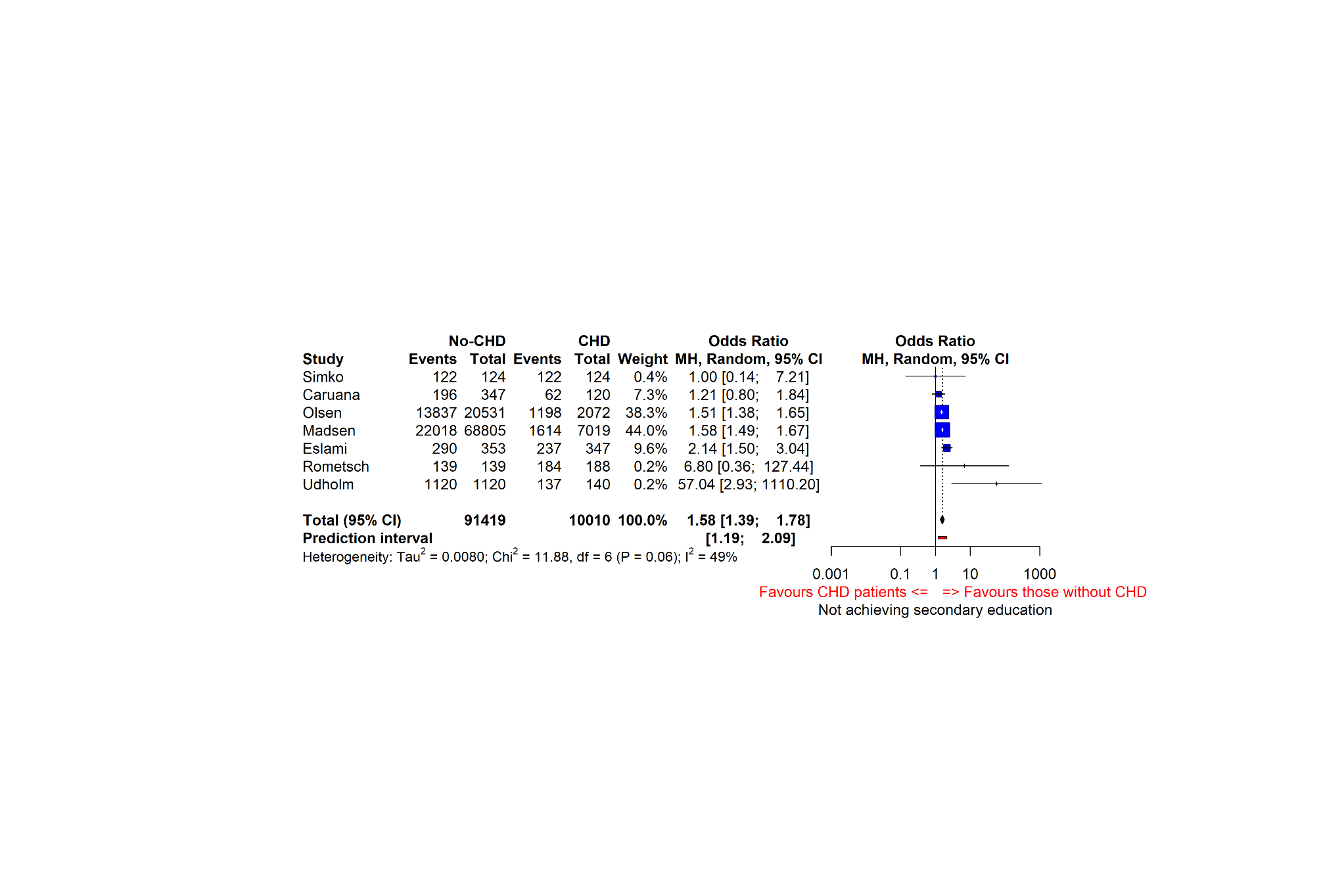


**Suplementary figure Figure 2c** **Pooled odds ratio of not achieving vocational training comparing CHD patients to those without CHD with control for age and sex**


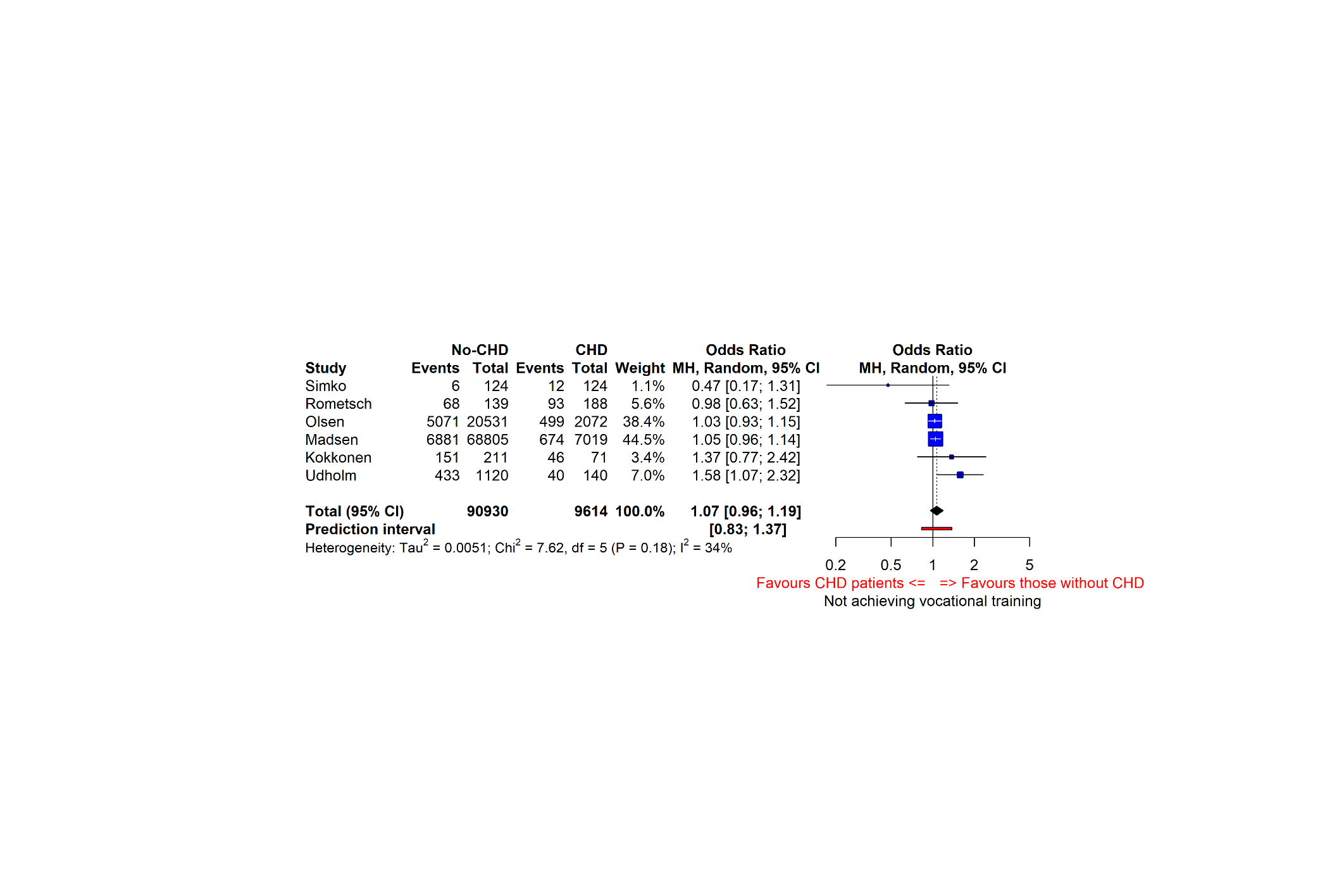


**Supplemenatry Figure 3a** **Pooled odds ratio of not achieving university degree comparing CHD patients to those without CHD including comparison with sibling**


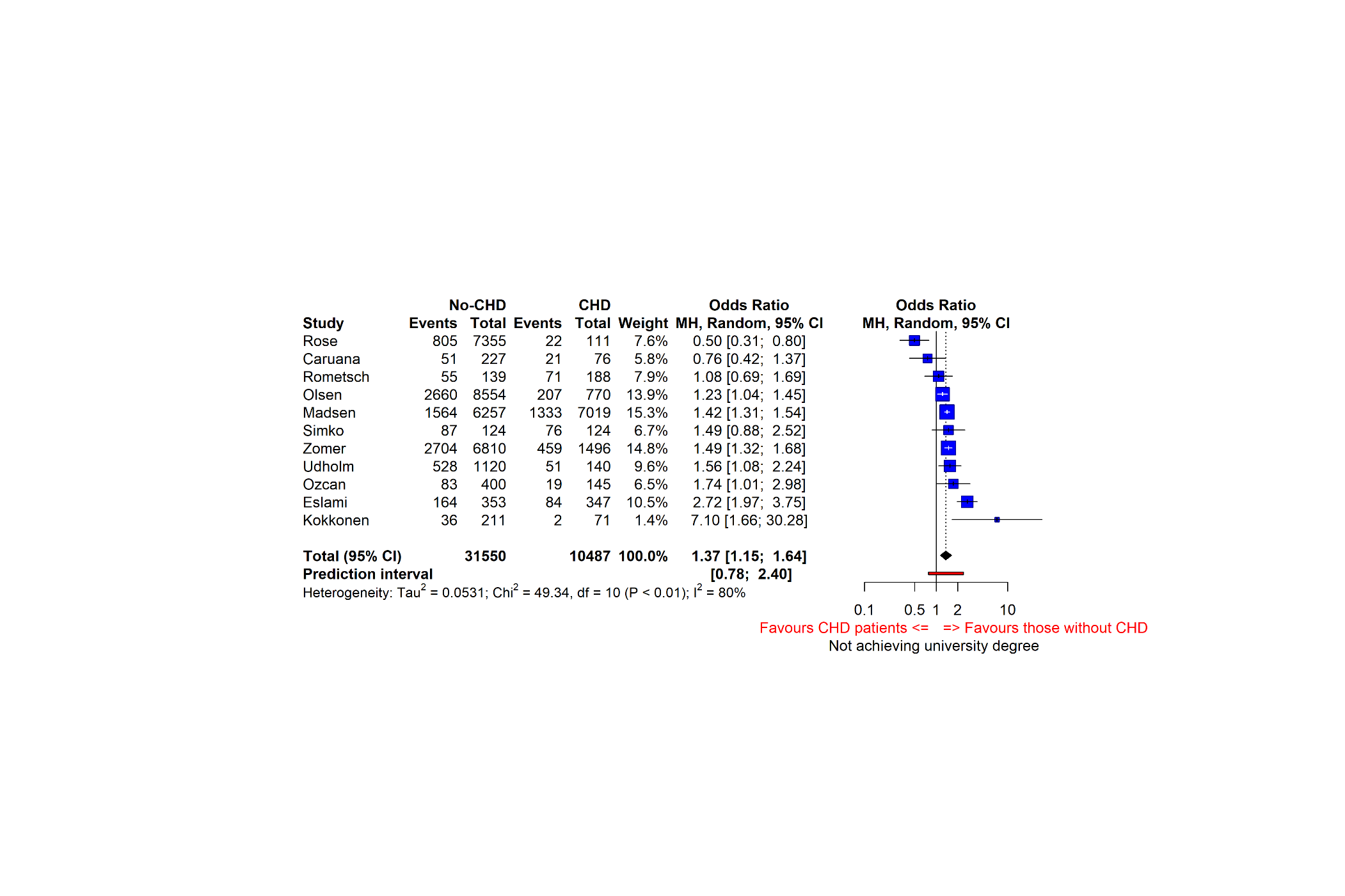


**Supplementary Figure 3b** **Pooled odds ratio of not achieving secondary educational attainment comparing CHD patients to those without CHD including comparison with sibling**


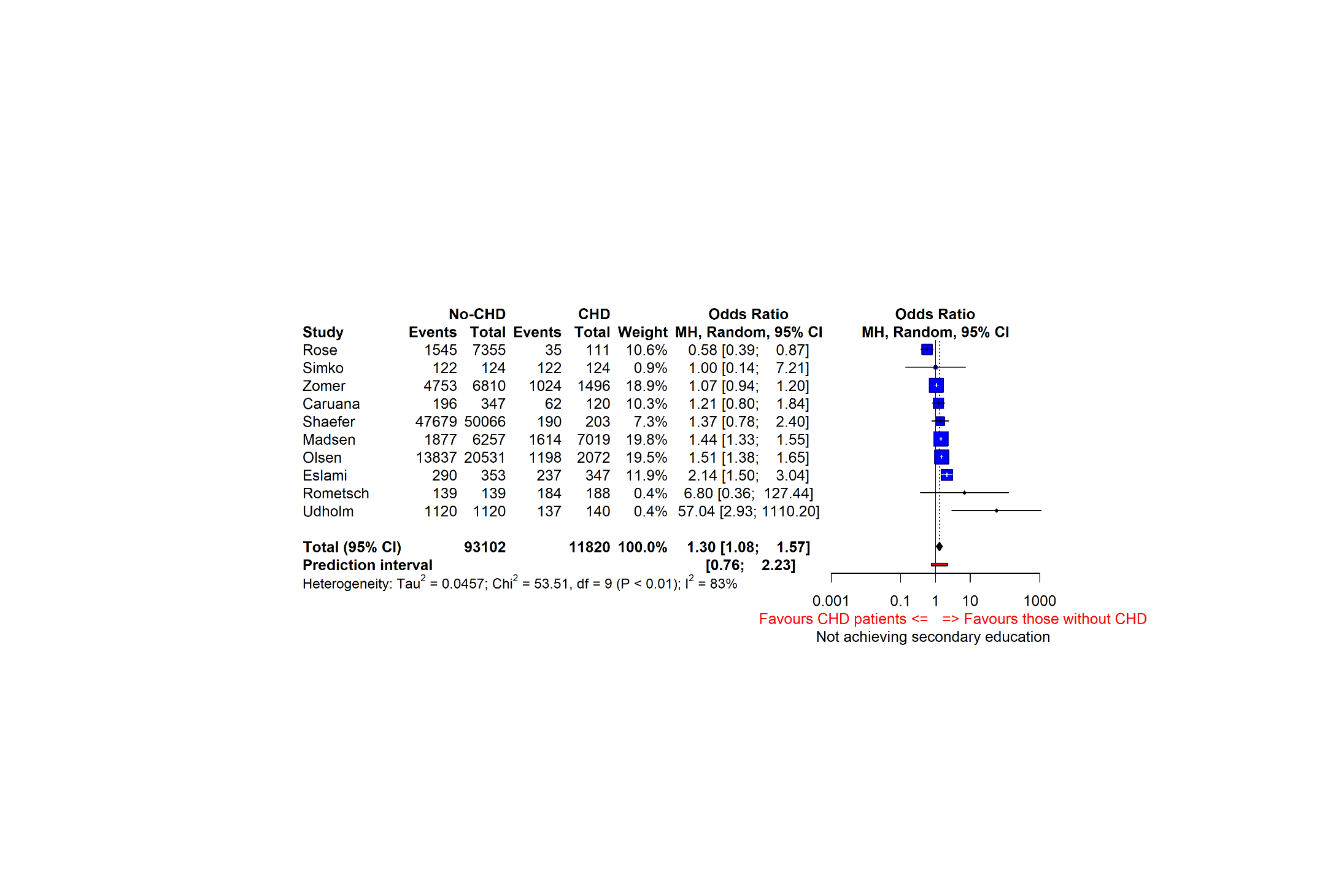


**Suplementary Figure 3c** **Pooled odds ratio of not achieving vocational training comparing CHD patients to those without CHD including comparison with sibling**


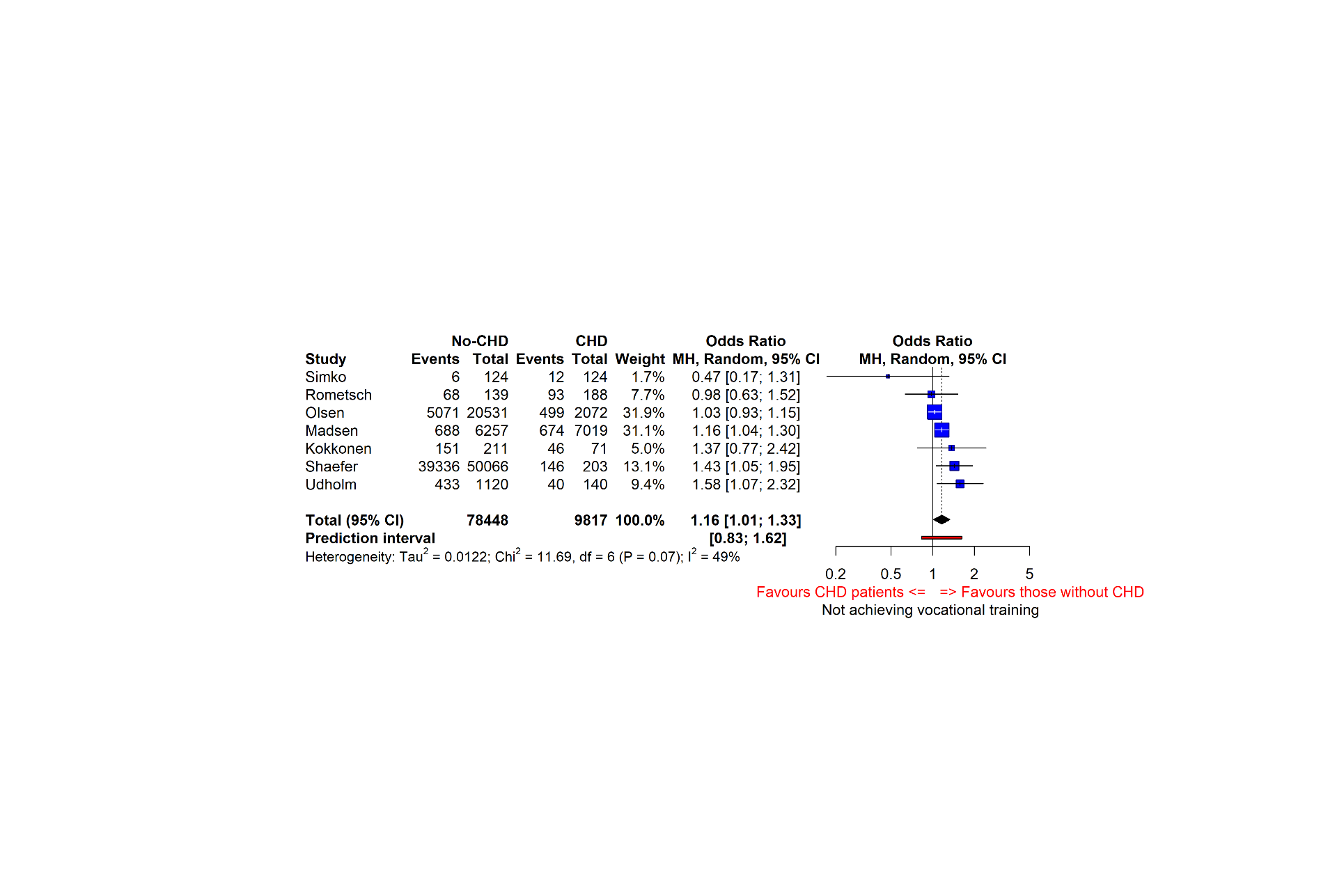


**Supplementary Figure 4a Pooled proportion of patients with CHD completing a university degree from all identified studies.**


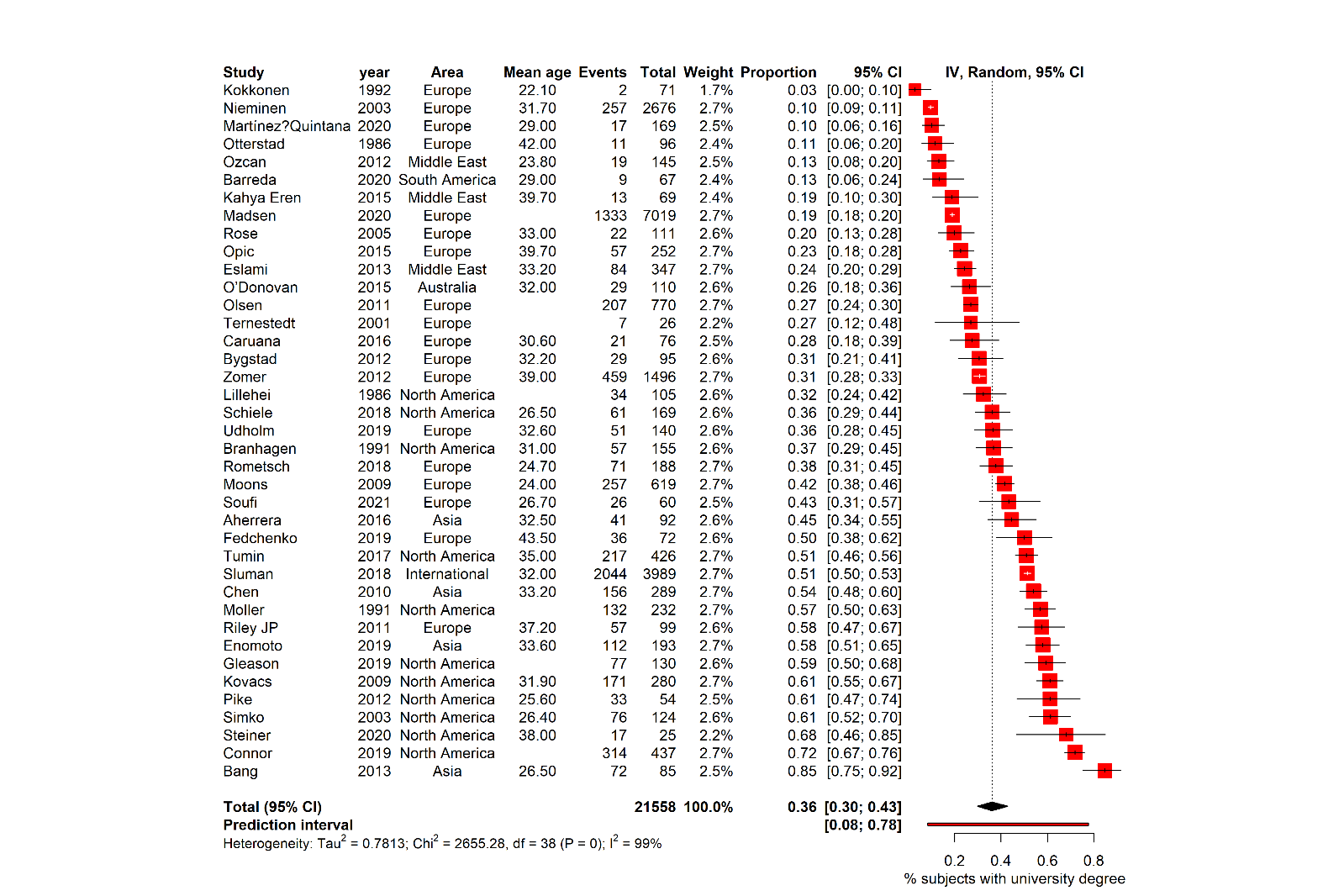


**Supplementary Figure 4b Pooled proportion of patients with CHD completing a secondary educational attainment from all identified studies**


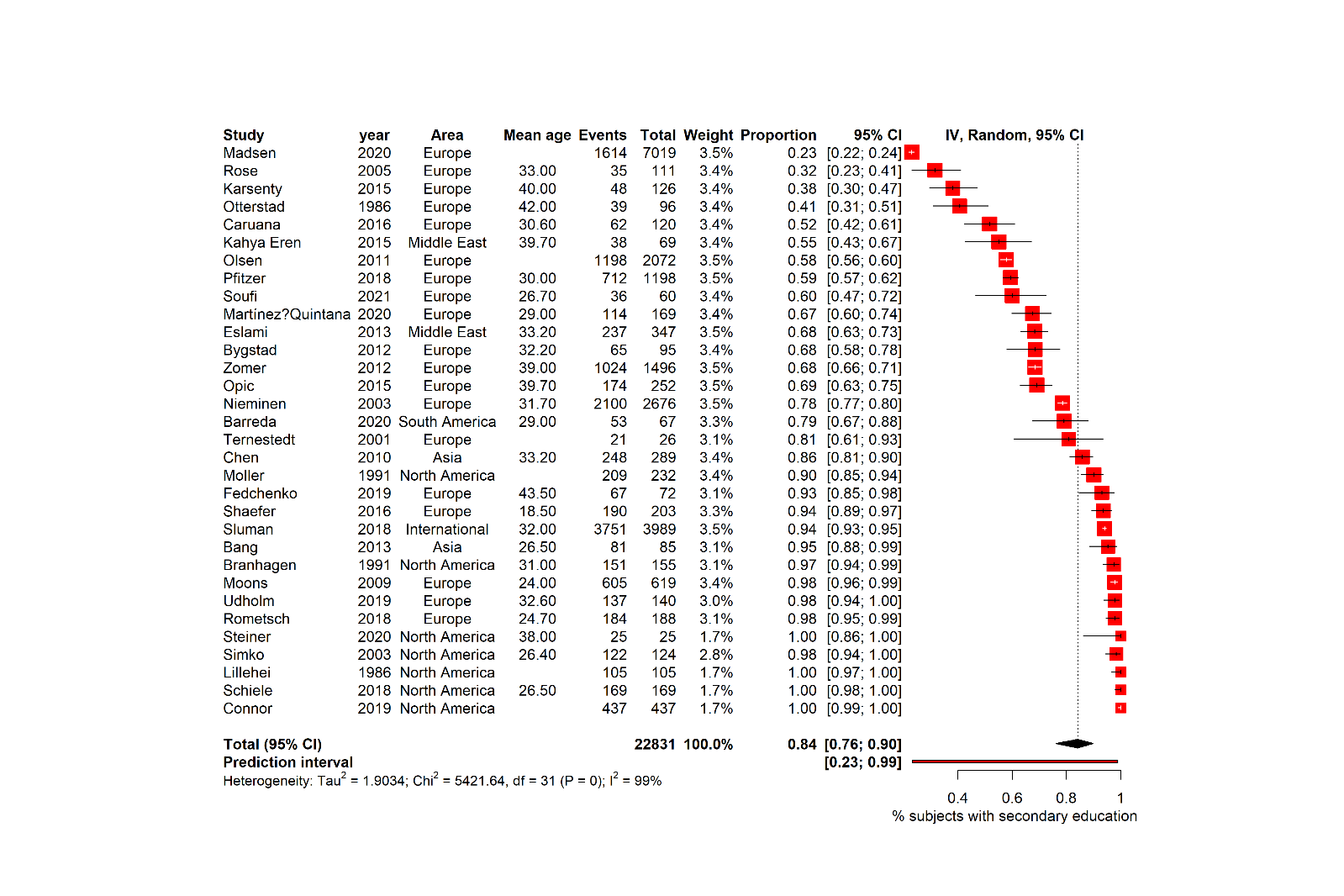


**Supplementary Figure 4c Pooled proportion of patients with CHD completing a vocational training from all identified studies**


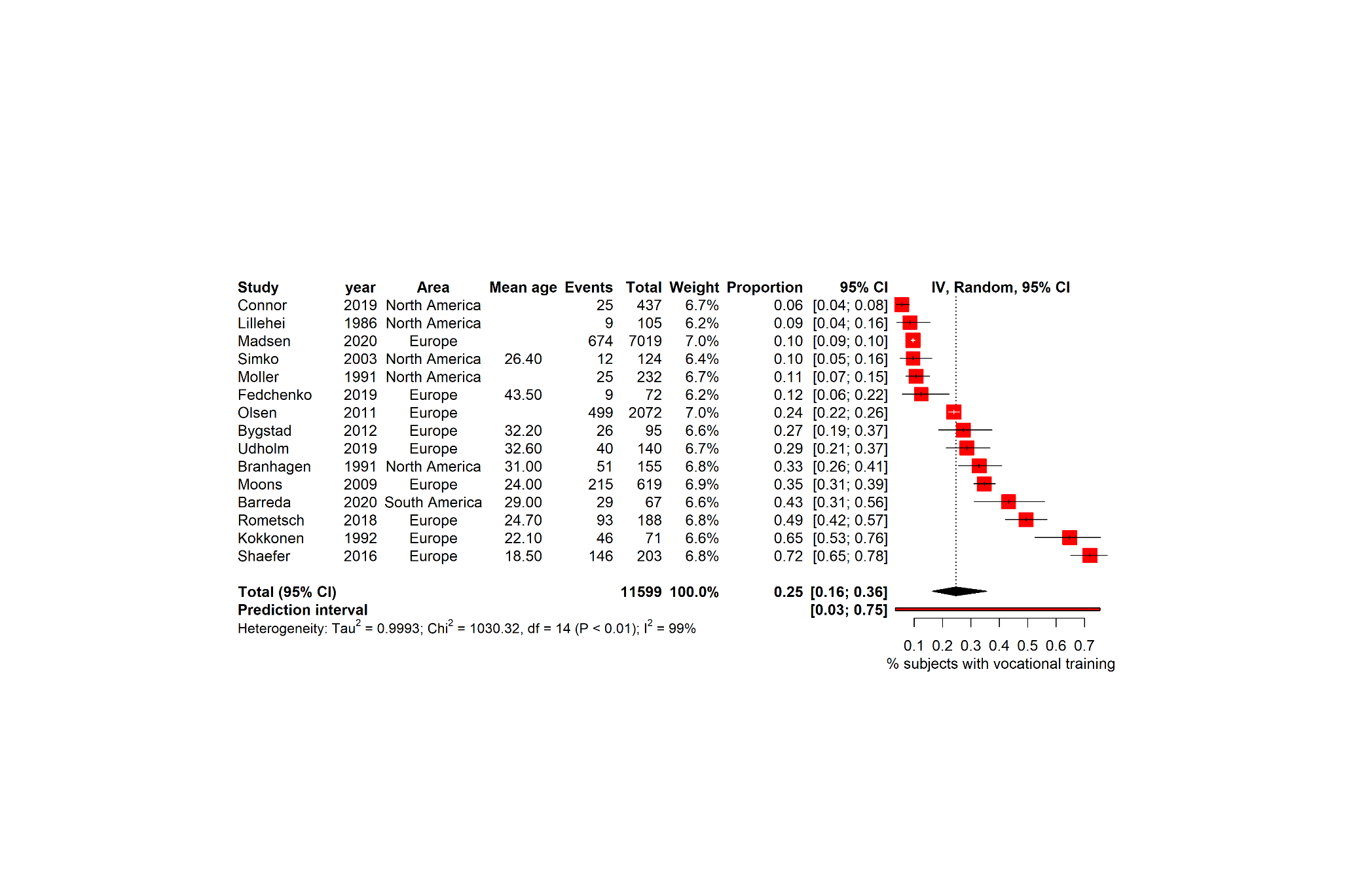


**Supplementary table 1. Overlapping studies excluded as overlapping with larger/more complete studies.**

| **Author, overlapping study** | **Study location** | **Study period** | **CHD population**  **Source** | **educational attainment data source** | **Number patients** | **Age** | **Type CHD** | **Control group** | **Study aims to assess education outcomes in patients with CHD** |
| --- | --- | --- | --- | --- | --- | --- | --- | --- | --- |
| Van Rijen Ehm (1)  Overlapping with Opic p, 2015(2) | Netherlands | First operation between 1968-1980 and recruitment 2000-2001 | The Department of Cardiothoracic surgery, Erasmus MC | Questionnaire | 362 | Mean 30.2 yrs range 20–46 | Mix CHD  20–33 years after their first heart surgery | normative data were derived from the Netherlands Central Bureau of Statistics | Yes |
| Sairanen Hi(3) overlapping with Nieminen H, 2003 (4) | Finland | Surgery between 1953-1989  And <15 years old, closing date 1998 | Finnish national research registry of pediatric cardiac surgery | Questionnaire | 2896 | Mean 27.6 (range 8.9-59 years) | Mix chd | Normative data from national statistical centre, Statistics Finland | Yes |
| Apers S(5)  Overlapping with Sluman MA, 2019(6) | International | 2013-2015 | Congenital Heart Disease-international study (APPROACH-IS) | Questionnaire | 3989 | Median 32 (IQR 25-42) | Mix CHD | - | No |
| Eaton SL(7)  Overlapping with Sluman MA, 2019(6) | Australia | 2013-2015 | Congenital Heart Disease-international study (APPROACH-IS) | Questionnaire | 135 | Median 25 (IQR 19-31) | Mix CHD | - | No |
| Vigl M(8) overlapping with  Pfitzer C, 2018(9) | Germany | - | National Registry for Congenital Heart Defects in Germany | Questionnaire | 1067 | Mean 32.1 (±14.4) | Mix CHD | 676 age and sex matched from the public use ﬁle of the German National Health Interview and Examination Survey | No |
| Bay A(10)  Overlapping with Sluman MA, 2019(6) | Sweden | 2013-2015 | Congenital Heart Disease-international study (APPROACH-IS) | Questionnaire | 471 | Mean 38 (±14.1) | Mix CHD | - | No |
| Moons P(11)  Overlapping with Sluman MA, 2019(6) | International | 2013-2015 | Congenital Heart Disease-international study (APPROACH-IS) | Questionnaire | 4028 | 32 (IQR 25–43) | Mix CHD | - | No |
| Pfitzer C  Overlapping with Pfitzer C, 2018(9) | Germany | Born 1992-2011 survey 2015 | German National Register for Congenital Heart Defects (NRCHD) | Questionnaire | 1198 | Mean 18.2 ± 4.4years | Mix CHD | Normative data German general population | Yes |

**Supplementary table 2 Tool for risk of bias in exposure studies(12)**

| **Author** | **Cofounding** | **Selection** | **Measurement of Exposure (CHD)** | **Departures from exposure** | **Missing data** | **Measurement OF**  **Outcome** | **Reported results** |
| --- | --- | --- | --- | --- | --- | --- | --- |
| Otterstad JE(13) | Low | Low | Low | Low | Low | Low | Low |
| Lillehei CW(14) | Low | Low | Low | Low | Low | Low | Low |
| Brandhagen DJ(15) | Low | Low | Low | Low | Serious | Low | High |
| Moller JH(16) | Low | Low | Low | Low | Serious | Low | Low |
| Kokkonen J(17) | Moderate | Low | Low | Low | Low | Moderate | Low |
| Ternestedt BM (18) | Low | Low | Low | Low | Serious | Low | Low |
| Nieminen H(4) | Low | low | Low | Low | Serious | Low | low |
| Simko LC(19) | Low | No information | Low | Low | Low | Moderate | Low |
| Rose M(20) | Critical | No information | Low | Low | Low | Low | Low |
| Kovacs AH(21) | Low | Low | Low | Low | Low | Low | Low |
| Moons P(22) | Low | Low | Low | Low | Low | Low | Low |
| Chen CA(23) | Low | Low | Low | Low | Low | Low | Low |
| Riley JP(24) | Low | Low | Low | Low | Low | Low | Low |
| Olsen M(25) | Low | Low | Low | Low | Low | Low | Low |
| Bygstad E(26) | Low | Low | Low | Low | Low | Low | Low |
| Pike NA(27) | Low | No information | Low | Low | Low | Low | Low |
| Ozcan EE(28) | Moderate | No information | Low | Low | Low | Low | Low |
| Zomer AC(29) | Critical | Serious | Low | Low | Low | Serious | Low |
| Eslami B (30) | Critical | No information | Low | Low | Low | Low | Low |
| Bang JS(31) | Low | No information | Low | Low | Low | Low | Low |
| Opic p(2) | Low | Serious | Low | Low | Low | Critical | Low |
| Karsenty C(32) | Low | Low | Low | Low | Low | Low | Low |
| Kahya Eren N(33) | Low | Low | Low | Low | Low | Serious | Low |
| O’Donovan CE(34) | Low | Serious | Low | Low | Low | Low | Low |
| Aherrera JAM(35) | Low | No information | Low | Low | Low | Low | Low |
| Schaefer CJ(36) | Critical | Low | Low | Low | Low | Low | Low |
| Caruana M(37) | Critical | Serious | Low | Low | Low | Low | Low |
| Tumin D(38) | Low | Moderate | Low | Low | Low | Low | Low |
| Gleason LP(39) | Low | Serious | Low | Low | Low | Low | Low |
| Schiele SE(40) | Low | Serious | Low | Low | Low | Low | Low |
| Rometsch S (41) | Critical | Low | Low | Low | Low | Low | Low |
| Udholm S(42) | Critical | Serious | Low | Low | Low | Low | Low |
| Fedchenko M(43) | Low | Critical | Low | Low | Low | Low | Low |
| Sluman MA(6) | Low | No information | Moderate | Low | Low | Low | Low |
| Pfitzer C(9) | Low | Serious | Low | Low | Low | Low | Low |
| Enomoto j(44) | Low | Serious | Low | Low | Low | Low | Low |
| Connor B(45) | Low | No information | Low | Moderate | Low | Low | Low |
| Madsen NL(46) | Low | Low | Low | Low | Low | Low | Low |
| Martínez‐Quintana(47) | Low | Low | Low | Low | Low | Low | Low |
| Steiner(48) | Low | Serious | Low | Low | Low | Low | Low |
| Berruda(49) | Low | Low | Low | Low | Low | Low | Low |
| Soufi(50) | Low | Low | Low | Low | Low | Low | Low |

**References**

1. Van Rijen EHM, Utens EMWJ, Roos-Hesselink JW, Meijboomb FJ, Van Domburg RT, Roelandt JRTC, et al. Psychosocial functioning of the adult with congenital heart disease: A 20-33 years follow-up. European Heart Journal. 2003;24(7):673-83.

2. Opić P, Roos-Hesselink JW, Cuypers JA, Witsenburg M, van den Bosch A, van Domburg RT, et al. Psychosocial functioning of adults with congenital heart disease: outcomes of a 30-43 year longitudinal follow-up. Clin Res Cardiol. 2015;104(5):388-400.

3. Sairanen HI, Nieminen HP, Jokinen EV. Late results and quality of life after pediatric cardiac surgery in Finland: a population-based study of 6,461 patients with follow-up extending up to 45 years. Seminars in thoracic and cardiovascular surgery. 2005;Pediatric cardiac surgery annual.:168-72.

4. Nieminen H, Sairanen H, Tikanoja T, Leskinen M, Ekblad H, Galambosi P, et al. Long-term results of pediatric cardiac surgery in Finland: education, employment, marital status, and parenthood. Pediatrics. 2003;112(6 Pt 1):1345-50.

5. Apers S, Kovacs AH, Luyckx K, Thomet C, Budts W, Enomoto J, et al. Quality of Life of Adults with Congenital Heart Disease in 15 Countries Evaluating Country-Specific Characteristics. Journal of the American College of Cardiology. 2016;67(19):2237-45.

6. Sluman MA, Apers S, Sluiter JK, Nieuwenhuijsen K, Moons P, Luyckx K, et al. Education as important predictor for successful employment in adults with congenital heart disease worldwide. Congenital Heart Disease. 2019;14(3):362-71.

7. Eaton SL, Wang Q, Menahem S. Determinants of quality of life in adults with CHD: an Australian cohort. Cardiol Young. 2017;27(8):1571-6.

8. Vigl M, Niggemeyer E, Hager A, Schwedler G, Kropf S, Bauer U. The importance of socio-demographic factors for the quality of life of adults with congenital heart disease. Quality of life research : an international journal of quality of life aspects of treatment, care and rehabilitation. 2011;20(2):169-77.

9. Pfitzer C, Helm PC, Rosenthal LM, Walker C, Ferentzi H, Bauer UMM, et al. Educational level and employment status in adults with congenital heart disease. Cardiol Young. 2018;28(1):32-8.

10. Bay A, Dellborg M, Berghammer M, Sandberg C, Engström G, Moons P, et al. Patient reported outcomes are associated with physical activity level in adults with congenital heart disease. Int J Cardiol. 2017;243:174-9.

11. Moons P, Luyckx K, Dezutter J, Kovacs AH, Thomet C, Budts W, et al. Religion and spirituality as predictors of patient-reported outcomes in adults with congenital heart disease around the globe. Int J Cardiol. 2019;274:93-9.

12. Morgan RL, Thayer KA, Santesso N, Holloway AC, Blain R, Eftim SE, et al. A risk of bias instrument for non-randomized studies of exposures: A users' guide to its application in the context of GRADE. Environ Int. 2019;122:168-84.

13. Otterstad JE, Tjore I, Sundby P. Social function of adults with isolated ventricular septal defects. Possible negative effects of surgical repair? Scand J Soc Med. 1986;14(1):15-23.

14. Lillehei CW, Varco RL, Cohen M, Warden HE, Gott VL, DeWall RA, et al. The first open heart corrections of tetralogy of Fallot. A 26-31 year follow-up of 106 patients. Ann Surg. 1986;204(4):490-502.

15. Brandhagen DJ, Feldt RH, Williams DE. Long-term psychologic implications of congenital heart disease: A 25-year follow-up. Mayo Clinic Proceedings. 1991;66(5):474-9.

16. Moller JH, Patton C, Varco RL, Lillehei CW. Late results (30 to 35 years) after operative closure of isolated ventricular septal defect from 1954 to 1960. Am J Cardiol. 1991;68(15):1491-7.

17. Kokkonen J, Paavilainen T. Social adaptation of young adults with congenital heart disease. International Journal of Cardiology. 1992;36(1):23-9.

18. Ternestedt BM, Wall K, Oddsson H, Riesenfeld T, Groth I, Schollin J. Quality of life 20 and 30 years after surgery in patients operated on for tetralogy of Fallot and for atrial septal defect. Pediatr Cardiol. 2001;22(2):128-32.

19. Simko LC, McGinnis KA. Quality of life experienced by adults with congenital heart disease. AACN clinical issues. 2003;14(1):42-53.

20. Rose M, Köhler K, Köhler F, Sawitzky B, Fliege H, Klapp BF. Determinants of the quality of life of patients with congenital heart disease. Qual Life Res. 2005;14(1):35-43.

21. Kovacs AH, Saidi AS, Kuhl EA, Sears SF, Silversides C, Harrison JL, et al. Depression and anxiety in adult congenital heart disease: predictors and prevalence. Int J Cardiol. 2009;137(2):158-64.

22. Moons P, Van Deyk K, Marquet K, De Bleser L, De Geest S, Budts W. Profile of adults with congenital heart disease having a good, moderate, or poor quality of life: a cluster analytic study. Eur J Cardiovasc Nurs. 2009;8(2):151-7.

23. Chen CA, Liao SC, Wang JK, Chang CI, Chiu IS, Chen YS, et al. Quality of life in adults with congenital heart disease: biopsychosocial determinants and sex-related differences. Heart. 2011;97(1):38-43.

24. Riley JP, Habibi H, Banya W, Gatzoulis MA, Lau-Walker M, Cowie MR. Education and support needs of the older adult with congenital heart disease. Journal of Advanced Nursing. 2012;68(5):1050-60.

25. Olsen M, Hjortdal VE, Mortensen LH, Christensen TD, Sørensen HT, Pedersen L. Educational achievement among long-term survivors of congenital heart defects: a Danish population-based follow-up study. Cardiol Young. 2011;21(2):197-203.

26. Bygstad E, Pedersen LC, Pedersen TA, Hjortdal VE. Tetralogy of Fallot in men: quality of life, family, education, and employment. Cardiol Young. 2012;22(4):417-23.

27. Pike NA, Evangelista LS, Doering LV, Eastwood JA, Lewis AB, Child JS. Quality of life, health status, and depression: comparison between adolescents and adults after the Fontan procedure with healthy counterparts. J Cardiovasc Nurs. 2012;27(6):539-46.

28. Ozcan EE, Kucuk A. Impact of severity of congenital heart diseases on university graduation rate among male patients. Turk Kardiyoloji Dernegi Arsivi. 2012;40(3):229-34.

29. Zomer AC, Vaartjes I, Uiterwaal CS, van der Velde ET, Sieswerda GJ, Wajon EM, et al. Social burden and lifestyle in adults with congenital heart disease. Am J Cardiol. 2012;109(11):1657-63.

30. Eslami B, Sundin O, Macassa G, Khankeh HR, Soares JJ. Anxiety, depressive and somatic symptoms in adults with congenital heart disease. J Psychosom Res. 2013;74(1):49-56.

31. Bang JS, Jo S, Kim GB, Kwon BS, Bae EJ, Noh CI, et al. The mental health and quality of life of adult patients with congenital heart disease. Int J Cardiol. 2013;170(1):49-53.

32. Karsenty C, Hascoet S, Blot-Souletie N, Galinier M, Maury P, Mondoly P, et al. The medical past of adults with complex congenital heart disease impacts their social development and professional activity. Archives of Cardiovascular Diseases. 2013;106 (8-9):469-70.

33. Eren NK, Kırdök AH, Kılıçaslan B, Kocabaş U, Düzel B, Berilgen R, et al. Quality of life of patients with atrial septal defect following percutaneous closure. Cardiol Young. 2015;25(1):42-6.

34. O'Donovan CE, Painter L, Lowe B, Robinson H, Broadbent E. The impact of illness perceptions and disease severity on quality of life in congenital heart disease. Cardiol Young. 2016;26(1):100-9.

35. Aherrera JAM, Abrahan LL, Racaza GZ, Train CQ, Jara RD. Depression and anxiety in adults with congenital heart disease using the validated filipino version of the hospital anxiety and depression score (HADS-P). Phillippine Journal of Internal Medicine. 2016;54(1).

36. Schaefer CJ, Hoop R, Schürch-Reith S, Stambach D, Kretschmar O, Bauersfeld U, et al. Academic achievement and satisfaction in adolescents with CHD. Cardiol Young. 2016;26(2):257-62.

37. Maryanne Caruana VG. Congenital Heart Disease has no Negative Impact on Educational Achievements and Employment among Maltese Adult Patients under Clinical Follow-Up. International Cardiovascular Forum Journal. 2016;8.

38. Tumin D, Chou H, Hayes D, Tobias JD, Galantowicz M, McConnell PI. Employment after heart transplantation among adults with congenital heart disease. Congenital Heart Disease. 2017;12(6):794-9.

39. Gleason LP, Deng LX, Khan AM, Drajpuch D, Fuller S, Ludmir J, et al. Psychological distress in adults with congenital heart disease: focus beyond depression. Cardiol Young. 2019;29(2):185-9.

40. Schiele SE, Emery CF, Jackson JL. The role of illness uncertainty in the relationship between disease knowledge and patient-reported outcomes among adolescents and adults with congenital heart disease. Heart Lung. 2019;48(4):325-30.

41. Rometsch S, Greutmann M, Latal B, Bernaschina I, Knirsch W, Schaefer C, et al. Predictors of quality of life in young adults with congenital heart disease. European Heart Journal - Quality of Care and Clinical Outcomes. 2019;5(2):161-8.

42. Udholm S, Nyboe C, Dantoft TM, Jørgensen T, Rask CU, Hjortdal VE. Small atrial septal defects are associated with psychiatric diagnoses, emotional distress, and lower educational levels. Congenit Heart Dis. 2019;14(5):803-10.

43. Fedchenko M, Mandalenakis Z, Dellborg H, Hultsberg-Olsson G, Bjork A, Eriksson P, et al. Cardiovascular risk factors in adults with coarctation of the aorta. Congenit Heart Dis. 2019;14(4):549-58.

44. Enomoto J, Mizuno Y, Okajima Y, Kawasoe Y, Morishima H, Tateno S. Employment status and contributing factors among adults with congenital heart disease in Japan. Pediatr Int. 2020;62(3):390-8.

45. Connor B, Osborne W, Peir G, Smith M, John A. Factors Associated With Increased Exercise in Adults With Congenital Heart Disease. American Journal of Cardiology. 2019.

46. Madsen NL, Marino BS, Woo JG, Olsen M. Comparison of Economic Self-Sufficiency and Educational Attainment in Adults With Congenital Heart Disease Versus Siblings Without Heart Disease and to General Population. Am J Cardiol. 2020;135:135-42.

47. Martinez-Quintana E, Girolimetti A, Jimenez-Rodriguez S, Fraguela-Medina C, Rodriguez-Gonzalez F, Tugores A. Prevalence and predictors of psychological distress in congenital heart disease patients. J Clin Psychol. 2020;76(9):1705-18.

48. Steiner JM, Dhami A, Brown CE, Stout KK, Curtis JR, Engelberg RA, et al. Barriers and Facilitators of Palliative Care and Advance Care Planning in Adults With Congenital Heart Disease. Am J Cardiol. 2020;135:128-34.

49. Lopez Barreda R, Guerrero A, de la Cuadra JC, Scotoni M, Salas W, Baraona F, et al. Poverty, quality of life and psychological wellbeing in adults with congenital heart disease in Chile. PLoS One. 2020;15(10):e0240383.

50. Soufi A, Gouton M, Metton O, Mitchell J, Bernard YF, Bozio A, et al. Quality of life of adult Fontan patients. Cardiol Young. 2021;31(1):97-104.
